# Biopathological Significance of PIWI–piRNA Pathway Deregulation in Invasive Breast Carcinomas

**DOI:** 10.3390/cancers12102833

**Published:** 2020-09-30

**Authors:** Didier Meseure, Sophie Vacher, Sabah Boudjemaa, Marick Laé, André Nicolas, Renaud Leclere, Walid Chemlali, Gabriel Champenois, Anne Schnitzler, Laetitia Lesage, Thierry Dubois, Ivan Bieche

**Affiliations:** 1 Unit of Pharmacogenomics, Department of Genetics, Institut Curie, 75005 Paris, France; sophie.vacher@curie.fr (S.V.); walid.chemlali@curie.fr (W.C.); anne.schnitzler@curie.fr (A.S.); ivan.bieche@curie.fr (I.B.); 2 Platform of Experimental Pathology PATHEX, Institut Curie, 75005 Paris, France; andre.nicolas@curie.fr (A.N.); renaud.leclere@curie.fr (R.L.); gabriel.champenois@curie.fr (G.C.); laetitia.lesage@curie.fr (L.L.); 3 Department of Diagnostic and Theranostic Medicine, Institut Curie, 75005 Paris, France; 4 Department of Pathology, Hôpital Armand Trousseau, 75012 Paris, France; sabah.boudjemaa@aphp.fr; 5 Department of Pathology, Centre Henri Becquerel, INSERM U1245 UniRouen Normandie Université, rue d’Amiens, 76000 Rouen, France; marick.lae@chb.unicancer.fr; 6 Breast Cancer Biology Group, Translational Research Department, Institut Curie, Université PSL, 75005 Paris, France; thierry.dubois@curie.fr; 7 INSERM U1016 Faculty of Pharmaceutical and Biological Sciences, Université Paris Descartes, 75005 Paris, France

**Keywords:** piRNA, PIWI proteins, transposable elements, silencing, methylation, heterochromatin, breast carcinomas

## Abstract

**Simple Summary:**

The PIWI-piRNA ribonucleoproteic complexes are pivotal regulators of genome integrity, differentiation and homeostasis and their dysregulation has recently been implicated in carcinogenesis. The aim of this study was to analyze the four PIWILs gene expression in invasive breast carcinomas (IBC) at RNA level using quantitative RT-PCR and protein level using immunohistochemistry. In normal breast tissue, PIWILs 2 and 4 were solely expressed, whereas an abnormal emergence of PIWIL1 and 3 was observed in respectively 30% and 6% of IBCs. Conversely, PIWIL2 was underexpressed in 48.3% and PIWIL4 downregulated in 43.3% of IBCs. Similar patterns of PIWIL deregulation were observed in a multitumoral panel, suggesting a generic mechanism in most cancers. PIWIL2 underexpression was significantly associated with DNA methylation and strong cytotoxic immune response. Characterization of the newly recognized PIWIL-piRNA pathway in IBCs opens interesting therapeutic perspectives using piRNAs, hypomethylating drugs, checkpoints immunotherapies and anti-PIWIL 1–3 antibodies.

**Abstract:**

The PIWI proteins emerging in the development of human cancers, edify PIWI-piRNA ribonucleoproteic complexes acting as pivotal regulators of genome integrity, differentiation and homeostasis. The aim of this study is to analyze the four PIWILs gene expression in invasive breast carcinomas (IBCs): at RNA level using quantitative RT-PCR (*n* = 526) and protein level using immunohistochemistry (*n* = 150). In normal breast tissue, PIWILs 2 and 4 were solely expressed, whereas an abnormal emergence of PIWIL1 and 3 was observed in respectively 30% and 6% of IBCs. Conversely, PIWIL2 was underexpressed in 48.3% and PIWIL4 downregulated in 43.3% of IBCs. Significant positive associations were observed between PIWIL4 underexpression, HR+ status and HR+ ERBB2+ molecular subtype and PIWIL2 underexpression, PR- status, ERBB2- status and molecular subtype. Similar patterns of PIWIL deregulation were observed in a multitumoral panel, suggesting a generic mechanism in most cancers. PIWIL2-4 underexpression was mainly regulated at epigenetic or post-transcriptional levels. PIWIL2 underexpression was significantly associated with DNA methylation and strong cytotoxic immune response. PIWIL2-4 were mainly associated with genes implicated in cell proliferation. As a result of this study, characterization of the PIWIL-piRNA pathway in IBCs opens interesting therapeutic perspectives using piRNAs, hypomethylating drugs, checkpoints immunotherapies and anti-PIWIL 1–3 antibodies.

## 1. Introduction

Among the heterogeneous group of invasive breast carcinomas (IBCs), triple-negative carcinomas (TNCs) remain a major cause of death in women. Identification of new prognostic factors and therapeutic targets is crucial to improve the outcome of this group of patients. Epigenetic abnormalities are early events in carcinogenesis associating global DNA hypomethylation with reactivation of transposable elements (TE), post-transcriptional modifications of histones and deregulation of noncoding RNAs (ncRNAs) [1,2]. PiRNAs (P-element induced wimpy testis (PIWI)-interacting RNAs), first identified in 2006, are small single-stranded ncRNAs of 25–33 nt characterized by their interaction with PIWI proteins that belong to the Argonaute family [3,4]. PIWI proteins are implicated in piRNAs biogenesis and edify ribonucleoproteins named PiRNA-induced silencing complexes (pi-RISCs) located into the cytoplasmic perinuclear “nuage” that recognize complementary targets and achieve RNA silencing at transcriptional and post-transcriptional levels [5] (Figure S1). PIWI proteins and piRNAs were first implicated in development, differentiation and maintenance of integrity and stability of germline cells through suppression of TEs activity [6,7,8,9]. However, mounting evidence has revealed that these are also instrumental in controlling gene expression—both in germinal and somatic cells—in germline stem-cell maintenance and self-renewal [10,11], development and fertilization [12,13,14,15], organogenesis and epigenetic activation [16,17,18], genes and proteins expression [19,20,21], brain maturation and plasticity [22,23], regeneration [24], pancreatic function [25] and fat metabolism [26]. Furthermore, accumulating data have suggested that they are implicated in tumorigenesis and associated with major hallmarks of cancer [27]. The four human PIWI proteins PIWIL1, PIWIL2, PIWIL3 and PIWIL4 can be included in the cancer/testis antigens (CTAs) class and their deregulation is involved in cancer cell proliferation, apoptosis and stemness [28,29,30,31,32,33,34], genomic integrity [27], invasion and metastasis [35,36,37,38]. More recently, PIWIL2 gene alternative variants directly produced from shorter mRNAs transcribed by intragenic promoters in the host gene of PIWIL2 have been implicated in carcinogenesis through their oncogenic properties [39,40]. Thus, deregulated PIWI proteins and their variants observed in somatic malignant tumors can represent potential diagnostic and prognostic biomarkers and pertinent targets of immunotherapy [41]. However, due to contradictory results in the literature, we investigated expression levels of the four human members of the PIWI family, both at RNA and protein levels by quantitative RT–PCR and immunohistochemistry (IHC) in a large series of 526 patients with IBC and in a multi-tumor panel composed of 16 different types of cancers.

## 2. Materials and Methods

### 2.1. Patients and Samples

Samples from 526 unilateral invasive primary breast tumors excised from women managed at Curie Institute, France, over a 30 years period (1978–2008) were analyzed (Table S1). Patients cared in our institution before 2007 were informed of the use of their samples for scientific purposes and had the opportunity to decline. Patients cared in our institution after 2007 signed an informed consent. This study was approved by the local ethics committee (Breast Group of Curie institute Hospital; Agreement number C75-05-18). Fresh samples were stored shortly after excision in liquid nitrogen until RNA extraction. A tumor sample was considered suitable for this study if the proportion of tumor cells exceeded 70%. All patients (mean age, 61.7 years; range, 31–91 years) met the following criteria: primary unilateral non metastatic breast carcinoma diagnosis for which complete clinical, histological and biologic data were available; no neoadjuvant radio or chemotherapy; and full follow-up at Curie Institute. Treatment consisted of modified radical mastectomy in 283 cases (63.9%) and breast-conservative surgery plus locoregional radiotherapy in 160 cases (36.1%). The patients had physical examination and routine chest radiographies every three months for two years and then annually. Mammograms were performed annually. Adjuvant therapy was administered to 369 patients, consisting in chemotherapy alone in 91 cases, hormone therapy alone in 176 cases and combined treatments in 102 cases. The histological subtype and the number of positive axillary lymph nodes were evaluated at the time of surgery. Infiltrating carcinomas were scored according to Scarff–Bloom–Richardson (SBR) histoprognostic system. Estrogen-receptor (ERα), progesterone-receptor (PR) and human EGF-receptor 2 (ERBB2) status was determined at the protein level using biochemical methods (dextran-coated charcoal method, enzyme immunoassay or immunohistochemistry (IHC)) and confirmed by real-time quantitative RT–PCR assays. The population was divided into 4 groups according to HR (Erα and PR) and ERBB2 status, as follows: two luminal subtypes (HR+ (Erα+ or PR+)/ERBB2+ (*n* = 58)) and (HR+ (Erα+ or PR+)/ERBB2- (*n* = 294)); an ERBB2+ subtype (HR- (Erα- and PR-)/ERBB2+ (*n* = 101)); and a triple-negative subtype (HR- (Erα- and PR-)/ERBB2- (*n* = 73)). Patients (*n* = 209) developed metastasis during a median follow-up of 8.9 years (range, 6 months to 29 years). Normal RNA sources were provided by 14 breast tissue specimen (morphologically normal breast tissue > 1 cm to infiltrating carcinomas (*n* = 7) and breast tissue from cosmetic surgery (*n* = 7)).

Samples from 150 IBCs and 10 normal breast tissues belonging to the large series of 526 IBCs were used to perform an IHC analysis of PIWIL1-2-3-4 proteins. The population was divided into 4 groups according to HR (Erα and PR) and ERBB2 status, as follows: two luminal subtypes (HR+ (Erα+ or PR+)/ERBB2+ (*n* = 15)) and (HR+ (Erα+ or PR+)/ERBB2- (*n* = 98)); one ERBB2+ subtype (HR- (Erα- and PR-)/ERBB2+ (*n* = 15)); and one triple-negative subtype (HR- (Erα- and PR-)/ERBB2- (*n* = 22)).

Samples from 30 pre-invasive breast lesions, including atypical columnar metaplasia (*n* = 10), atypical ductal hyperplasia (*n* = 10) and ductal carcinoma in situ (*n* = 10), were used to perform IHC analysis of PIWIL1-2-3-4 proteins.

Samples from 16 primary malignant tumors of various subtypes (*n* = 628) and 16 normal various tissues (*n* = 249) were collected for RNA extraction and RT–PCR analysis from multicenter Departments of Pathology from 1978 to 2010 (Table S2).

Samples from (i) normal testicular tissues (*n* = 10), (ii) 17 somatic normal tissues (*n* = 160) and (iii) 17 primary malignant tumors (*n* = 170) were collected from multicenter Departments of Pathology from 2005 to 2015 to evaluate semiquantitatively the four PIWIL proteins using IHC.

### 2.2. RNA Extraction

Total RNA was extracted from breast tumor samples using acid-phenol guanidium as previously described [42]. RNA quality was determined by electrophoresis through agarose gels, staining with ethidium bromide and visualization of the 18S and 28S RNA bands under ultraviolet light.

### 2.3. Real-Time RT–PCR

PIWIL1, PIWIL2, PIWIL3 and PIWIL4 gene mRNA expression levels were quantified using real time RT–PCR. Quantitative values were obtained from the cycle number (*C*_t_ value) at which the increase in the fluorescence signal associated with exponential growth of PCR products started to be detected by the laser detector of the ABI Prism 7900 sequence detection system (Perkin-Elmer Applied Biosystems, Thermo Fisher Scientific, Inc., Waltham, MA, USA), using PE Biosystems analysis software according to the manufacturer’s manuals. The precise amount of total RNA added to each reaction mix (based on optical density) and its quality (i.e., lack of extensive degradation) are both difficult to assess. We therefore also quantified transcripts of the TBP gene (GenBank accession: NM_003194) encoding the TATA box–binding protein (a component of the DNA-binding protein complex TFIID) as an endogenous RNA control and normalized each sample on the basis of its TBP content. We selected TBP as endogenous control because the prevalence of its transcripts is moderate and because there are no known TBP retropseudogenes (retropseudogenes lead to co-amplification of contaminating genomic DNA and thus interfere with RT–PCR, despite the use of primers in separate exons). Results, expressed as *N*-fold differences in target gene expression relative to the TBP gene and termed “*N*_target_,” were determined as *N*_target_ = 2^Δ*C*t_*sample*^, where the Δ*C*_t_ value of the sample was determined by subtracting the average *C*_t_ value of the target gene from the average *C*_t_ value of the TBP gene. For each tumor type, the *N*_target_ values of the samples were subsequently normalized such that the median of the *N*_target_ values for 14 normal tissues was 1. The smallest amount of mRNA that was detectable and quantifiable by RT-qPCR (∆Ct = 35) was used as a reference (basal mRNA level = 1) to normalize the data for normal and tumoral tissue samples. For each tumor type, the *N*_target_ values of the samples were also subsequently normalized such that the median of the *N*_target_ values for each normal tissue type was 1. *N*_target_ values of 0.33 or less were considered to represent underexpression and values of 3 or more to represent overexpression of these genes in tumor samples. We previously used the same approach to determine cutoff points for the altered expression of tumor genes [43]. The primers for TBP, PIWIL1, PIWIL2, PIWIL3 and PIWIL4 genes were chosen with the assistance of the Oligo 6.0 program (National Biosciences, Plymouth, MN, USA). We scanned the dbEST and nr databases to confirm the total gene specificity of the nucleotide sequences chosen for the primers and the absence of SNPs (Table S3). To avoid amplification of contaminating gDNA, one of the two primers was placed at the junction between 2 exons or on 2 different exons. Agarose gel electrophoresis was used to verify the specificity of PCR amplicons. The conditions of cDNA synthesis and PCR were as described [44].

### 2.4. Immunohistochemistry

Immunohistochemistry (IHC) assay was performed using PIWIL1 (R&D System AF6548, goat polyclonal, 1/50 1 h, pH 6, Minneapolis, MN, USA), PIWIL2 (Novusbio NBP2-41126, rabbit polyclonal, 1/800, 1 h, pH 9, Centennial, CO, USA), PIWIL3 (Novusbio NBP1-76490, rabbit polyclonal, 1/500, 1 h, pH 6), PIWIL4 (Novusbio NBP2-37398, mouse monoclonal 10G9B11, 1/400, 1 h, pH 9), CD8 (DAKO M7103, monoclonal mouse C8/144B, 1/100, 30 min, pH 9, Carpinteria, CA, USA), DNMT1 (Abcam Ab134148, monoclonal rabbit EPR3521(2), 1/50, 1 h, pH 9, Cambridge, UK), histone H1 (Abcam Ab11079, monoclonal mouse, 34, 1/15000, 15 min, pH 9), HP1a (Abcam Ab109028, polyclonal rabbit, 1/500, 15 min, pH 9), SUV39H1 (Bethyl A302-127A, polyclonal rabbit, 1/400, 1 h, pH 9, Montgomery, TX 77356, USA), H3K9me3 (Abcam Ab8898, polyclonal rabbit, 1/700, 15 min, pH 9) and H3K27me3 (Cell Signaling #9733, monoclonal rabbit C36B11, 1/200, 1 h, pH 6, Beverly, MA, USA) antibodies. Paraffin-embedded tissue blocks, obtained at the time of the initial diagnosis, were retrieved from the archives of the Department of Diagnostic and Theranostic Medicine, Curie Institute. Sections of 3 μm in thickness were cut with a microtome from the paraffin-embedded tissue blocks of normal breast tissue, pre-invasive lesions and IBCs. Tissue sections were deparaffinized and rehydrated through a series of xylene and ethanol washes. Briefly, key figures included: (i) antigen retrieval in 0.1 mol/L citrate buffer, pH 6 (BioCare, Pacheco, CA, USA) in a pressure cooker (4 min); (ii) blocking of endogenous peroxidase activity by immersing sections in 3% hydrogen peroxide in methanol for 15 min a nd subsequently rinsing them in water and PBS; (iii) incubation with primary antibodies against the targeted antigen; (iv) immunodetection with a biotin-conjugated secondary antibody formulation that recognizes rabbit and mouse immunoglobulins, followed by peroxidase-labeled streptavidin and linking with a rabbit biotinylated antibody against mouse immunoglobulin G (DAKO SA) and (v) chromogenic revelation with DAB and counterstaining with Mayer’s hematoxylin. All immunostainings were processed using a Leica BOND RX research automated immunostaining device. Antibodies specificity was confirmed by a panel of human tissues containing lymphocytes. A semi quantitative histological score (HScore = intensity × frequency) was used for interpretation (0 = negative staining, 1 = weak staining, 2 = moderate staining and 3 = strong staining).

### 2.5. Statistical Analysis

The distributions of target mRNA levels were characterized by their median values and ranges. Relationships between mRNA levels of the different target genes, mRNA (and protein) levels and clinical parameters were identified using nonparametric tests, namely, the χ² test (relation between 2 qualitative parameters), the Mann–Whitney U test (relation between 1 qualitative and 1 quantitative parameter) and the Spearman rank correlation test (relation between 2 quantitative parameters). Differences were considered significant at confidence levels up to 95% (*p* < 0.05). Metastasis-free survival (MFS) was determined as the interval between initial diagnosis and detection of the first metastasis. Survival distributions were estimated by the Kaplan–Meier method. The significance of differences between survival rates was ascertained with the log rank test [45].

### 2.6. In Silico DNA Methylation Data and mRNA Expression Levels Analysis

DNA methylation and mRNA expression levels in the TCGA breast tumor series (*n* = 1108) used for statistical analysis were downloaded from the publicly available database cBioPortal for Cancer Genomics (https://www.cbioportal.org) [44,46].

## 3. Results

### 3.1. The Purely Germinal or Mixed Germinal and Somatic Status of the Genes Encoding the PIWIL1-2-3-4 Proteins Depends on the Origin of Tissues

We compared PIWIL1-2-3-4 genes expression status in testicular germ cells to somatic cells belonging to a panel of 16 various normal tissues (*n* = 249) at RNA level. Expression status of the four PIWIL proteins were also evaluated semiquantitatively using IHC in a panel of testicular tissues (*n* = 10) and various somatic normal tissues (*n* = 160). PIWIL1, PIWIL2, PIWIL3 and PIWIL4 genes were strongly expressed by normal testicular germ cells and weaker by somatic cells of various normal tissues.

In normal testicular germinal cells, all four human homologs of PIWIL genes were strongly expressed at RNA and protein levels. By using RT–PCR, the 4 PIWIL genes presented high levels of expression (PIWIL1 vs. TBP vs. *Ct35* = 1:28800, PIWIL2:10458, PIWIL3:182 and PIWIL4:2207). Through IHC, PIWIL1 had cytoplasmic immunostaining of moderate intensity (HScore: 2) and low intensity nuclear staining (HScore: 1) in the more mature cells (spermatids and spermatozoa). PIWIL2 presented cytoplasmic immunostaining of very low intensity (HScore: 0 to 0.5) and moderate to high intensity nuclear staining (HScore: 2 to 3) in germ cells at different maturation stages (spermatogonia, spermatids and spermatozoa). PIWIL3 had low to moderate cytoplasmic intensity immunostaining (HScore: 1 to 2) and low to moderate intensity nuclear immunostaining (HScore: 1 to 2) in mature cells (spermatids and spermatozoa). PIWIL4 had weak cytoplasmic immunostaining (HScore: 1) and variable nuclear staining (HScore: 1 to 3) present in germ cells at various maturation stages (spermatogonia (HScore: 1 to 2) spermatids (HScore: 1 to 2) and spermatozoa (HScore: 3)) (Figure S2A).

In most human normal tissues, a multitissue panel (*n* = 16) was used to evaluate expression levels of PIWIL1-2-3-4. We identified two expression profiles comparable at RNA and protein levels. By using RT–PCR, the first profile associating lack of PIWIL1 and PIWIL3 expression and weak expression of PIWIL2 and PIWIL4 was observed in head and neck, prostate, bladder, liver, pancreas, lung, cervix, stomach and anal canal normal tissues. The second profile associating lack of expression of PIWIL3 and weak expression of PIWIL1, PIWIL2 and PIWIL4 was identified in kidney, brain, colon, ovary, thyroid and endometrial normal tissues (Table 1; Figure S6a,b). By using IHC, the first profile was also observed in the same tissues with slight immunostaining (HScore: 1) of PIWIL2 and PIWIL4 in respiratory head and neck mucosal, prostate, liver, pancreas, bladder, cervix, lung, stomach and anal canal normal tissues. The second profile characterized by weak expression (HScore: 1) of PIWIL1, PIWIL2 and PIWIL4 was observed in kidney, brain, endometrium, ovary, colon and thyroid normal tissues (Table 2).

### 3.2. In Breast Tissue, only Mixed Somatic and Germinal Genes Encoding PIWIL2 and PIWIL4 Proteins Are Normally Expressed and Thus Participate in piRNAs Biogenesis and PIWI–piRNA Pathway Activity

By comparison with normal testicular germinal cells, normal breast tissues presented a characteristic profile combining absence of PIWIL1 and PIWIL3 expression (pure germinal status) and PIWIL2 and PIWIL4 low expression (mixed germinal and somatic status) at RNA and protein levels.

By using RT–PCR, PIWIL1-2-3-4 genes presented variable levels of expression (PIWIL1:0 (0–7.87), PIWIL2:66.8 (28.5–118), PIWIL3:0 (0–0) and PIWIL4:138 (60.2–118)) (Table 1).

Through immunohistochemistry, PIWIL1 immunostaining was absent in epithelial and myoepithelial cells. PIWIL2 presented a strong nuclear staining (HScore: 3) in myoepithelial cells and a moderate intensity nuclear staining (HScore: 2) in epithelial cells with a very slight cytoplasmic staining (HScore: 0–0.5). Like PIWIL1, PIWIL3 was not expressed in epithelial and myoepithelial cells. PIWIL4 presented a slight to moderate nuclear staining (HScore: 1–2) both in myoepithelial and epithelial cells and a very slight cytoplasmic staining (HScore: 0–0.5) (Figure S2B).

### 3.3. IBCs Are Characterized by Deregulation of All Proteins PIWIL1-2-3-4 Levels of Expression

We determined expression levels of PIWIL1-2-3-4 genes in patients with IBCs.

**Figure 1:**
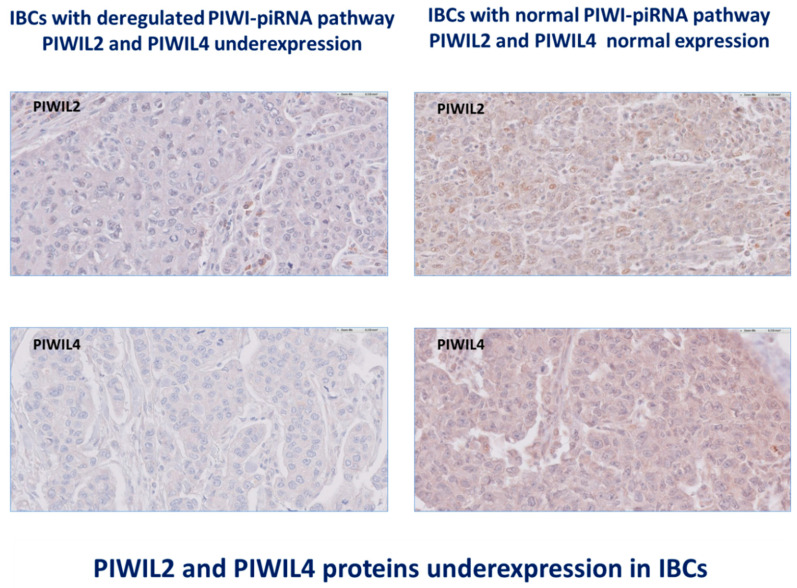

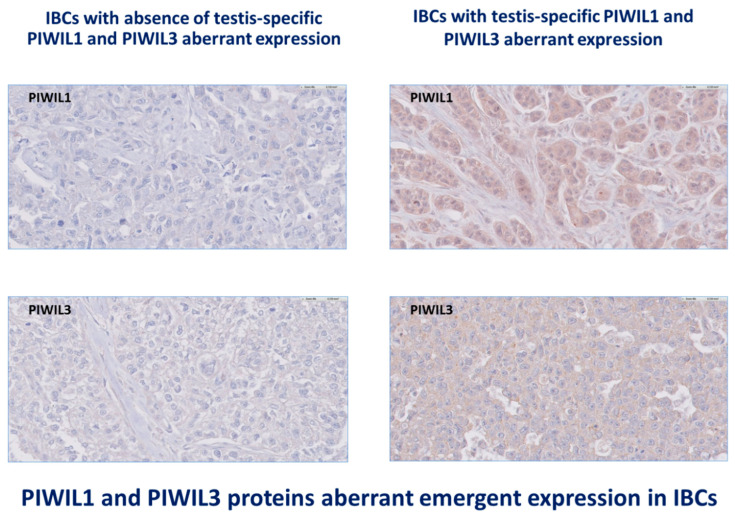
Invasive breast carcinomas (IBCs) are characterized by deregulation of all proteins PIWIL1-2-3-4. At magnification ×200.

Comparisons with samples from normal breast tissues revealed that in our series of 526 IBCs, all four human PIWIL genes homologs were expressed both at RNA level (PIWIL1:0 (ranged from 0 to 15.53), PIWIL2:0.34 (ranged from 0 to 19.38), PIWIL3:0 (ranged from 0 to 151.97) and PIWIL4:0.37 (ranged from 0 to 2.96)) (Table 3) and at protein level (PIWIL1, HScore: 0–1; PIWIL2, HScore: 0.5–1; PIWIL3, HScore: 0–1 and PIWIL4, HScore: 0.5–1). PIWIL1, PIWIL2, PIWIL3 and PIWIL4 were all deregulated with a dissociated profile: PIWIL1 and PIWIL3 had an abnormal emergent expression and the remaining PIWIL2 and PIWIL4 were variably downregulated in tumor tissues at RNA and protein levels (Figure 1).

We measured mRNA levels of PIWIL1-2-3-4 using quantitative RT–PCR assays, in a series of 526 unilateral invasive breast tumors and 14 normal breast tissues (adjacent to breast cancer or collected from cosmetic breast surgery specimen).

The mRNA values of the breast cancer samples were normalized such that the median of the 14 normal breast tissue mRNA values was 1. Values of 0.33 or less were considered to represent underexpression and values of 3 or more to represent overexpression of these genes in tumor samples. We have previously used the same approach to determine cutoff points for tumor gene altered expression [47].

**Figure 2:**
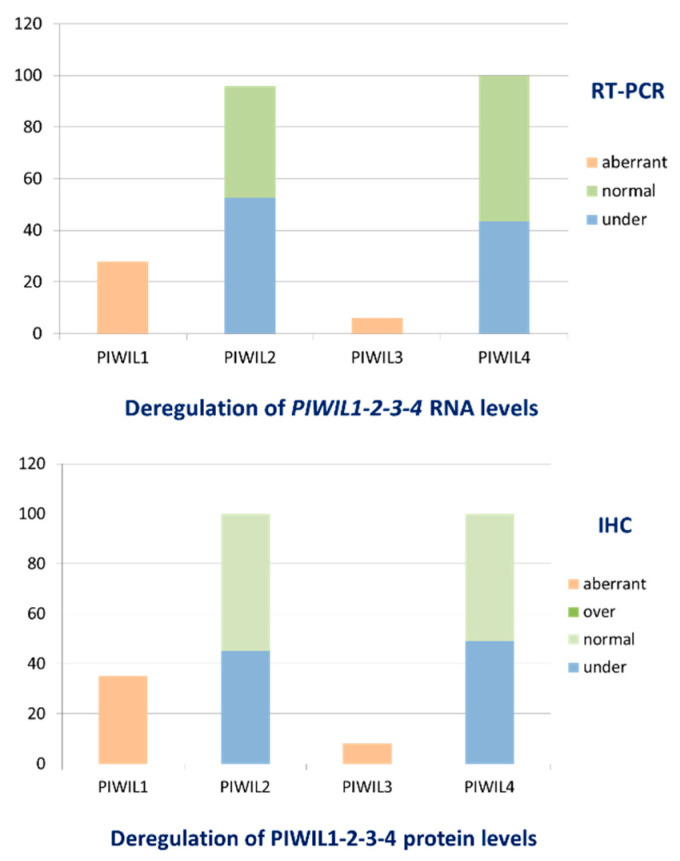
IBCs are characterized by deregulation of all proteins PIWIL1-2-3-4 at RNA and protein levels.

In our series of 526 IBCs, we observed abnormal emerging expression of PIWIL1 in 30.2% (*n* = 159) of IBCs and PIWIL3 in 5.9% (*n* = 32) of IBCs. Conversely, PIWIL2 was underexpressed in 48,3% (*n* = 254) of IBCs and PIWIL4 downregulated in 43,3% (*n* = 228) of IBCs (Figure 2, Table 3). Furthermore, we identified a statistically moderate significant positive correlation between PIWIL2 and PIWIL4 mRNA expression levels (*p* < 0.0001) in IBCs (Figure S4).

PIWIL2 and PIWIL4 mRNA expression was correlated with amount of PIWIL2 and PIWIL4 proteins in IBCs. A statistically significant correlation was identified between PIWIL2/4 mRNA and PIWIL2/4 protein expression levels (PIWIL2 (*p* = 0.0019) and PIWIL4 (*p* < 0.0001)), suggesting that PIWI genes deregulation could mainly be transcriptional in IBCs (Table S4).

Regarding molecular subtypes of IBCs, we subdivided our total population of IBCs (*n* = 526) into four subgroups: HR+/ERBB2+ (*n* = 58), HR+/ERBB2- (*n* = 294), HR-/ERBB2+ (*n* = 73) and HR-/ERBB2- (*n* = 101) and we observed few differences between molecular subtypes. PIWIL1 aberrant emerging expression and PIWIL2 underexpression were observed in all subgroups. Aberrant emerging expression of PIWIL3 was observed more frequently in HR-HER2+ tumors (15%) than in HR-/ERBB2- tumors (3%) and in HR+HER2- tumors (4%). PIWIL4 underexpression was identified more frequently in HR+HER2+ tumors (53.4%) than in HR-HER2+ tumors (39.7%) and in HR-/HER2- tumors (28.7%) (Table 3).

We performed IHC analysis using anti-PIWIL1, PIWIL2, PIWIL3 and PIWIL4 antibodies in a series of 150 IBCs (out of 526) and 10 normal breast tissues.

PIWIL1-2-3-4 proteins are expressed both in epithelial cancer cells and in different subpopulations of tumor microenvironment in IBCs. IHC assay confirmed PIWIL1-2-3-4 proteins localization in breast epithelial cancer cells and several subtypes of stromal and inflammatory/immune cells of the tumor microenvironment. Carcinoma cells exhibited a nuclear and/or cytoplasmic staining with various intensity. PIWIL1 staining was exclusively observed in the cytoplasm of cancer cells with a slight intensity (HScore: 1). PIWIL2 staining was identified in the nucleus of cancer cells with a slight intensity (HScore: 0.5–1). PIWIL3 was located both in the nucleus and the cytoplasm of cancer cells with a slight intensity (HScore: 1). PIWIL4 staining was also observed in the cytoplasm (HScore: 1) and in the nucleus (HScore: 0–1). Stromal cells, including immune cells, endothelial cells and fibroblasts were identified with anti-PIWIL1, anti-PIWIL2 and anti-PIWIL4 antibodies (HScore: 0.5–3) and used as internal positive controls (Figure 1).

We identified immunostaining of tumor cells with anti-PIWIL1 antibodies in 34,6% of IBCs (*n* = 52) and anti-PIWIL3 antibodies in 8% of IBCs (*n* = 12) and absence or slight staining with anti-PIWIL2 antibody in 48,3% of IBCs (*n* = 67) and anti-PIWIL4 antibody in 48,6% of IBCs (*n* = 73) (Figure 2).

Regarding molecular subtypes of IBCs, we subdivided our total population of IBCs (*n* = 150) into four subgroups: HR+/ERBB2+ (*n* = 15), HR+/ERBB2- (*n* = 98), HR-/ERBB2+ (*n* = 15) and HR-/ERBB2- (*n* = 22). We also observed few differences between molecular subtypes by using IHC with PIWIL3 aberrant expression and PIWIL4 underexpression more frequently observed in HR-HER2+ tumors (26%) and HR+HER2- tumors (45%), respectively (Table S6).

### 3.4. The Oncogenic Variant PL2L60 of the Oncosuppressor PIWIL2 Is Underexpressed in IBCs

Recent data identified variants of PIWIL2, including *PL2L80, PL2L80A, PL2L60, PL2L60A, PL2L50* and *PL2L40,* that are not derived from canonical splicing of full length mRNAs of PIWIL2 gene, but directly produced from short form of mRNAs transcribed by intragenic promoters located in the host gene of PIWIL2. Interestingly, while full length PIWIL2 acts as an oncosuppressor via DNA repair, the *PL2L60* variant seems to have oncogenic properties in tumor progression [39,40]. We measured mRNA level of *PL2L60* in our large series of breast cancer and observed underexpression of this variant in 59% of IBCs and overexpression in 3% of IBCs. In HR-/ERBB2- tumors, *PL2L60* was underexpressed in 63.7% of IBCs with absence of overexpression, in HR-ERBB2+ tumors, *PL2L60* was downregulated in 74% and upregulated in 1% of IBCs, in HR+/ERBB2- tumors, *PL2L60* was underexpressed in 56% and overexpressed in 4% of IBCs and in HR+/ERBB2+ tumors, *PL2L60* was underexpressed in 49% with overexpression in 8% of IBCs.

### 3.5. Similar Patterns of PIWIL1-2-3-4 Abnormal Levels of Expression Are Observed in a Multitumoral Panel, Suggesting a Generic Mechanism Initiating PIWI–piRNA Pathway Deregulation in Cancers

Because of contradictory data in the recent literature regarding expression levels of PIWIL1-2-3–4 proteins in IBCs and other malignant tumors, a multitumor panel (*n* = 16) was analyzed to identify expression status of PIWIL1-2-3-4 genes at RNA level in most human cancers. By using RT–PCR, mixed germinal–somatic PIWIL2 and PIWIL4 genes were more frequently downregulated in endometrial, bladder, prostate, skin, colon, ovary, cervix, head and neck and anal canal carcinomas. PIWIL1 gene was overexpressed in colon (95.9%), stomach (31.0%), ovary (28.8%), cervix (27.0%), thyroid (25.8%) and anal canal tumors (12.5%). PIWIL3 gene had emerging aberrant levels of expression in bladder (18.4%), endometrial (17.2%), ovary (11.5%), lung (11.1%) and liver (6.5%) tumors and was overexpressed in skin (3.7%) and lung (1.9%) tumors (Table S7). By using IHC, mixed germinal–somatic PIWIL2 and PIWIL4 proteins were downregulated than normal tissues in endometrial, bladder, pancreas, brain, stomach, colon, ovary, cervix, head and neck and anal canal carcinomas. Germinal PIWIL3 protein was not expressed in any malignant tumor belonging to the multitumor panel (Table 2).

### 3.6. Deregulation of the PIWIL1-2-3-4 Proteins Is Early during Breast Carcinogenesis

In order to determine whether PIWI proteins are also deregulated at an early step of breast carcinogenesis, we analyzed PIWIL1-2-3-4 expression by IHC in a series of 10 normal breast tissues and 20 pre-invasive neoplastic lesions, including atypical ductal hyperplasia (*n* = 10) and ductal carcinoma in situ (*n* = 10). We observed progressive PIWIL2 and PIWIL4 downregulation with absence or very slight nuclear staining (HScore = 0–0.5) in atypical ductal hyperplasia (PIWIL2 (30%, *n* = 3) and PIWIL4 (20%, *n* = 2)) and ductal carcinoma in situ (PIWIL2 (40%, *n* = 4) and PIWIL4 (40%, *n* = 4)) (Figure S3). PIWIL1 showed slight cytoplasmic immunostaining (HScore: 1) in atypical ductal hyperplasia (10%, *n* = 1 and ductal carcinoma in situ (10%, *n* = 1). PIWIL3 did not present any nuclear or cytoplasmic immunoreactivity in atypical ductal hyperplasia and ductal carcinoma in situ.

### 3.7. PIWIL1-2-3-4 mRNA Expression Levels Are Correlated with Classical Clinicopathological Factors

Significant positive associations were observed between the tumor group showing PIWIL4 underexpression and HR+ status (*p* = 0.0002) and HR+ ERBB2+ molecular subtype (*p* = 0.0037). In the tumor group showing PIWIL2 underexpression, significant positive associations were observed with PR- status (*p* = 0.0025), ERBB2 negative status (*p* = 0.039) and molecular subtype (*p* = 0.004). In the tumor group showing PIWIL1 emerging expression, significant positive associations were observed with lymph node status (*p* = 0.0065) and macroscopic tumor size (*p* = 0.21). In the tumor group showing PIWIL3 emerging expression, significant positive associations were observed with PR- status (*p* = 0.026), ERBB2-negative status (*p* = 0.0004) and molecular subtype (*p* = 0.0017) (Table S5).

To further investigate whether PIWIL1-2-3-4 mRNA expression could be of prognosis relevance, the log-rank test was used to identify the link between MFS (metastasis-free survival) and PIWIL1-2-3-4 mRNA expression. Unlike other recent results of the literature, we did not observe any significant association between PIWIL1/PIWIL3 emerging expression or PIWIL2/PIWIL4 downregulation and MFS (PIWIL1, *p* = 0.84; PIWIL2, *p* = 0.18; PIWIL3, *p* = 0.94; PIWIL4, *p* = 0.36) (Figure S5a) and between PIWIL1/PIWIL3 emerging expression or PIWIL2/PIWIL4 downregulation and OS (PIWIL1, *p* = 0.50; PIWIL2, *p* = 0.97; PIWIL3, *p* = 0.22; PIWIL4, *p* = 0.25) (Figure S5b).

### 3.8. PIWIL2-4 Underexpression Is Related to Epigenetic or Post-Transcriptional Mechanisms

**Figure 3:**
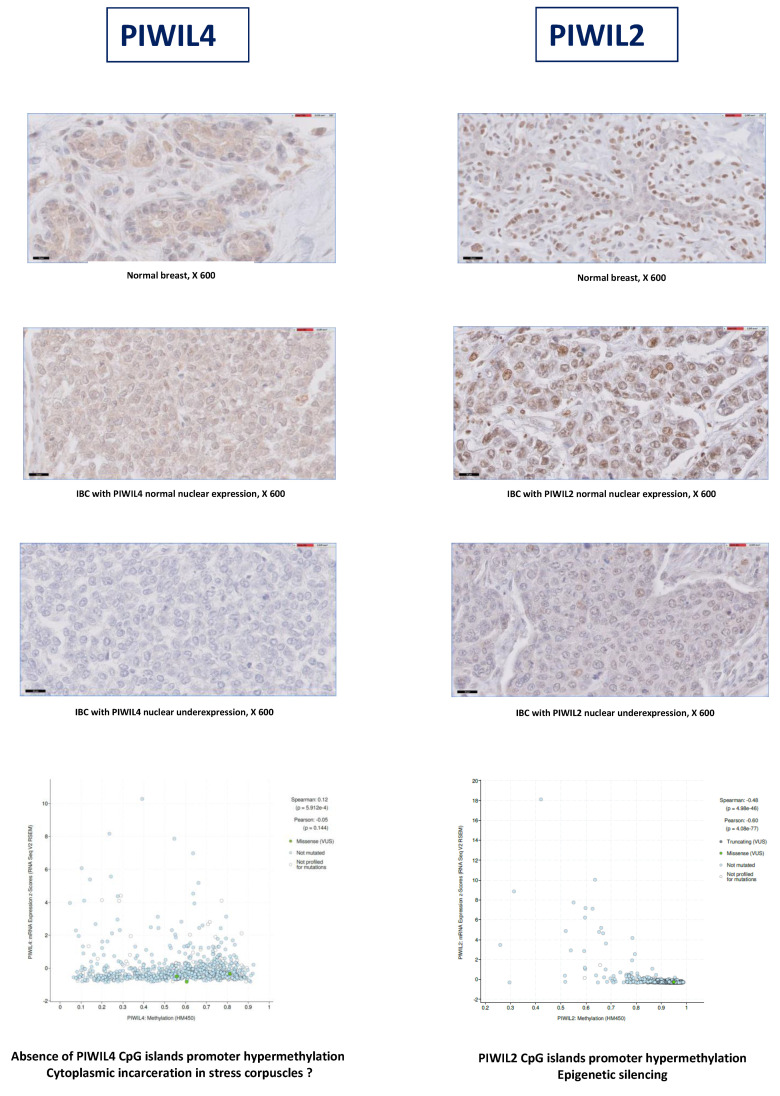
In silico and IHC analysis suggests that mechanisms of PIWIL2-4 underexpression are located at epigenetic or post-transcriptional levels. At magnification ×600.

We performed a combined IHC and in silico analysis of PIWIL2 and PIWIL4 mRNA levels of expression and methylation status from the publicly available database cBioPortal for The Cancer Genomics Atlas (TCGA) (https://www.cbioportal.org) [45,46]. We observed that IBCs with PIWIL2 underexpression had a hypermethylation status of its promoter, whereas IBCs with PIWIL2 normal expression had a normal methylation status, suggesting a transcriptional and epigenetic silencing. Regarding PIWIL4, the lack of correlation between its level of expression and the methylation status of its promoter suggests an inactivating mechanism other than epigenetic (Figure 3).

### 3.9. PIWIL2 Underexpressing IBCs Are Significantly Correlated at Protein Level with Downregulation of Histone H1, DNMT1, HP1 and SUV39H1 Proteins and Upregulation of CD8 Cytotoxic Immune Reaction

**Figure 4:**
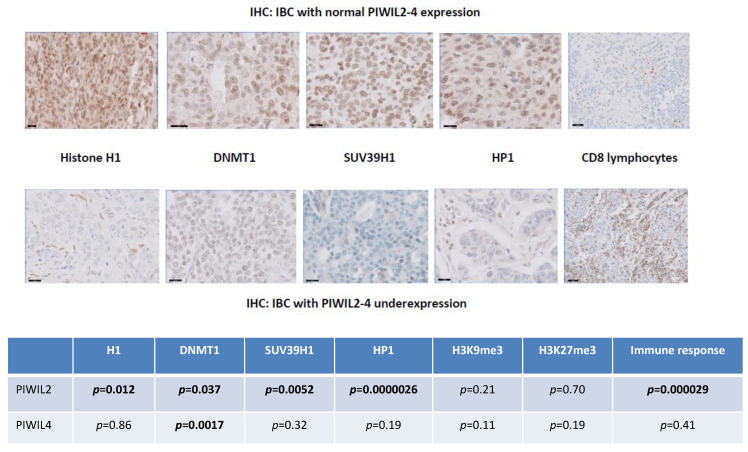
PIWIL2-4 underexpression is correlated with downregulation of H1, DNMT1, HP1 and SUV39H1 and upregulation of CD8 cytotoxic immune response. At magnification ×800.

We compared PIWIL2 and PIWIL4 proteins expression to immunohistochemical staining scores of CD8, DNMT1, H1 histone, HP1, SUV39H1, H3K9me3 and H3K27me3 (χ² test) in our series of 150 IBCs. IBCs with PIWIL2 underexpression were characterized by DNA and histone methyltransferase downregulation (histone H1, *p* = 0.012; DNMT1, *p* = 0.037; HP1, *p* = 0.0000026; SUV39H1, *p* = 0.0052) whereas IBCs with PIWIL4 underexpression were only characterized by DNA methyltransferase DNMT1 underexpression (*p* = 0.0017). There was no significant correlation with H3K9me3 and H3K27me3. By analyzing tumor microenvironment, we could observe that IBCs with PIWIL2 underexpression (but not PIWIL4 underexpression) were significantly correlated with increased immune cytotoxic CD8+ response (*p* = 0.000029) (Figure 4).

### 3.10. PIWIL2 and PIWIL4 Genes Are Associated with Hallmarks of Cancer

Recent literature data suggested that PIWI proteins were associated with cancer hallmarks. We explored the possible correlation of the PIWI–piRNA signaling pathways with well-known genes implicated in carcinogenesis. PIWIL2 and PIWIL4 downregulation in IBCs of our series were positively associated with genes implicated in cell cycle and proliferation (i.e., *PLK1* and *AURKA*) with an inverse correlation with genes acting in EMT (i.e., *VIM*, *TWIST1* and *JUB*), stemness (i.e., *ALDH1A1*, *ALDH1A3* and *CD133*), genome integrity (i.e., *ATM*), oncogenic pathways (i.e., RTK (*PDGFRA*, *DDR2*, *PDGFRB*, *IGF2*, *EGFR*), MAPK (*ETV5, ETV1*) and PI3K (*AKT3, FOXO1, FOXO4, PIK3R1, IRS2*)), angiogenesis (i.e., *VEGFC*, *PTGS2*) and migration (i.e., *EHD2*, *NCAM1* and *CAV1*) (Table S8).

## 4. Discussion

The four human PIWI proteins, i.e., PIWIL1, PIWIL2, PIWIL3 and PIWIL4, constitute the catalytic components of the pi-RISCs complexes implicated in piRNA biogenesis and are guided by piRNAs to specific targets based on sequence specific complementarity. They are considered as epigenetic regulators functioning at transcriptional and post-transcriptional levels [48,49]. In our series, PIWIL1-2-3-4 expression status at RNA and protein levels of testicular germ cells was compared to somatic cells belonging to normal breast tissues and various other normal tissues. We observed that status of genes encoding PIWIL1-2-3-4 proteins depended on tissues origin. PIWIL1-2-3-4 RNAs and proteins were strongly expressed by normal germ cells and weaker by somatic cells of various normal tissues. We identified two expression profiles comparable at RNA and protein levels. The first profile associating lack of PIWIL1 and PIWIL3 expression and weak expression of PIWIL2 and PIWIL4 (PIWIL1- PIWIL2+ PIWIL3- PIWIL4+ profile) was observed in head and neck, prostate, bladder, liver, pancreas, lung, cervix, stomach and anal canal normal tissues. The second profile associating weak expression of PIWIL1, PIWIL2 and PIWIL4 and lack of expression of PIWIL3 (PIWIL1+ PIWIL2+ PIWIL3- PIWIL4+ profile) was identified in kidney, brain, endometrium, ovary, colon and thyroid normal tissues. The first profile was observed in normal breast tissue of our series. The comparison of PIWIL1-2-3-4 genes expression levels in germinal and somatic tissues aimed to specify their germinal or mixed status. PIWIL3 gene was only expressed in normal germ cells (pure germinal status) whereas PIWIL2 and PIWIL4 genes were expressed in normal germinal and somatic tissues (mixed germinal and somatic status). PIWIL1 gene had germinal or mixed status according to the origin of tissues.

Since discovery of the unexpected role of the PIWI–piRNA pathway in seminoma, both in vivo and in vitro functional studies combined with small clinicopathological analysis have identified all four human PIWI proteins as new molecular players in various types of cancers [50,51,52,53,54]. PIWI proteins were thus found to be involved in most of cancer hallmarks, including cell proliferation, apoptosis, invasion and metastasis and could represent diagnostic and prognostic biomarkers [33,36,37,38,55,56]. However, data concerning both expression levels of PIWIL1-2-3-4 proteins and their prognostic and predictive values were contradictory in the small series reported in the literature [56,57]. We identified a generic deregulation profile of the four PIWI proteins in most of cancers analyzed. We observed in our large series of IBCs that PIWIL1-2-3-4 were all deregulated with a particular profile associating abnormal emerging expression of germinal PIWIL1 and PIWIL3 with downregulation of PIWIL2 and PIWIL4 both at RNA and protein levels. Significant positive correlation was identified between PIWIL2/4 mRNA and protein expression levels (PIWIL2 (*p* = 0.0007) and PIWIL4 (*p* < 0.0001)), suggesting that *PIWI* genes expression could be in part dysregulated at transcriptional level in IBCs. Hypermethylation of PIWIL2 promoters CpG islands was suggested by identification of the transcriptional level of PIWIL2 regulation, absence of PIWIL2 cytoplasmic location of this protein in IBCs with PIWIL2 protein underexpression and a hypermethylation status of PIWIL2 promoter in IBCs with PIWIL2 underexpression using IHC and in silico analysis, respectively. Regarding PIWIL4, lack of correlation between its level of expression and methylation status of its promoter suggests another mechanism of inactivation (Figure S5). These results are in partial agreement with several mechanisms of deregulation recently mentioned in the literature, such as epigenetic inactivation of the PIWIL2 gene by hypermethylation of promoters CpG islands and delocalization of PIWI proteins into cytoplasmic stress bodies [58]. PIWIL2 variants constitute a complex regulatory network that could partly explain versatility of PIWI proteins and contradictory results in the literature. While full length PIWIL2 functions as an oncosuppressor promoting DNA repair and apoptosis, PL2L60 protein overexpression could promote tumorigenesis through upregulating several signal transduction pathways and inhibiting apoptotic death of tumor cells [39,40]. Unlike data in the literature, we observed in our large series that PL2L60 was deregulated, but underexpressed in 59% of IBCs with an overexpression in only 3% of IBCs. IHC assay confirmed that PIWIL1-2-3-4 proteins were localized within breast epithelial tumor cells. PIWIL1 was exclusively observed in the cytoplasm of tumor cells and PIWIL2 was identified in the nucleus. PIWIL3 and PIWIL4 were located both in the nucleus and the cytoplasm of tumor cells. PIWIL1, PIWIL2 and PIWIL4 were also localized within the nucleus and the cytoplasm of several subtypes of cells belonging to the tumor microenvironment such as immune cells, endothelial cells and fibroblasts. Interestingly, alteration of the PIWI proteins expression appears early during breast carcinogenesis. PIWIL2 and PIWIL4 were downregulated in pre-invasive breast lesions such as atypical ductal hyperplasia (PIWIL2 (30%) and PIWIL4 (20%)) and ductal carcinoma in situ (PIWIL2 (40%) and PIWIL4 (40%)). Positive associations were observed between IBCs with PIWIL4 underexpression and HR+ status and HR+ ERBB2+ molecular subtype whereas IBCs with PIWIL2 underexpression showed significant correlations with PR- status, ERBB2 status and molecular subtype, suggesting two different mechanisms of deregulation in breast cancers. However, in opposition to other recent results from the literature [57], we did not observe any significant association between *PIWIL1/PIWIL3* emerging expression or *PIWIL2/PIWIL4* downregulation and MFS.

Because we found contradictory data in the literature, a multitumor panel of 16 malignant tumor types was analyzed in order to identify expression status of PIWIL1-2-3-4 genes in human cancers at RNA and protein levels. In most of malignant tumors belonging to this panel, we observed an expression profile comparable to IBCs associating variable PIWIL2 and PIWIL4 genes downregulation and an emerging aberrant expression of PIWIL1 and PIWIL3. PIWIL1 gene had emerging aberrant levels in all types of tumors belonging to the panel and was overexpressed in colon, stomach, ovary, cervix, thyroid and anal canal tumors. *PIWIL3 gene* had emerging aberrant levels of expression in bladder, endometrial, ovary, lung and liver tumors and was only overexpressed in skin tumors. A second profile of expression associated variable PIWIL2 and PIWIL4 genes downregulation, an aberrant level of PIWIL1 and absence of PIWIL3 expression observed in anal canal, head and neck, cervix, skin, kidney and pancreas tumors. These two patterns of expression in 17 types of malignant tumors suggest a generic mechanism resulting in PIWI–piRNA pathway deregulation and inactivation in cancers.

The human genome includes various defense strategies to inactivate propagation and mobility of TEs, such as epigenetic repression by DNA methylation. In normal germline stem cells, piRNAs cooperate with abundant PIWI proteins to regulate TEs inactivation via DNA methylation [59,60,61]. Oncogenic progression is frequently accompanied by profound loss of DNA methylation which is a well-recognized hallmark of cancer. These epigenetic dysregulation mechanisms in tumor cells altering PIWI–piRNA pathway-induced control of TEs propagation lead to extensive TEs expression, which in turn alter both tumor cells integrity and immune response. In this regard, we compared PIWIL2 and PIWIL4 RNA levels of expression to protein levels of expression of molecules involved in chromatin accessibility and genome methylation such as DNA methyltransferase DNMT1, H1 histone, HP1, SUV39H1, H3K9me3 and H3K27me3. We observed that IBCs with PIWIL2/PIWIL4 underexpression were significantly associated with downregulation of DNMT1, H1, HP1 and SUV39H1. IBCs with PIWIL2 underexpression were characterized by DNMT1, histone H1, HP1 and SUV39H1 downregulation whereas IBCs with PIWIL4 underexpression were only associated with DNMT1 downregulation. By analyzing the tumor microenvironment, we could observe that IBCs with PIWIL2 underexpression were significantly correlated with increased immune cytotoxic CD8+ response, suggesting that PIWIL2 could constitute a pertinent predictive biomarker of sensitivity to immunotherapies.

The oncogenic or tumor-suppressing status of the PIWI–piRNA pathway remains poorly understood. Because of their frequent underexpression, PIWI proteins implicated in biogenesis of piRNAs could be considered as oncosuppressors that would lead to PIWI–piRNA-induced deregulated control of many oncogenes. Experimental data have identified several mechanisms implicated in acquisition of cancer hallmarks defined by Weinberg and Hanahan [51,52,62]. We observed that carcinomas with inactivation of the PIWI–piRNA pathway were significantly associated with genes implicated in proliferation with an inverse correlation with genes acting in EMT, stemness and migration.

Recent studies revealed the potential for piRNAs as therapeutic tools and PIWI proteins as targets in cancers. In our series, we could observe aberrant emerging expression of PIWIL1 in 30% and PIWIL3 in 6% of IBCs, respectively. Data from the literature show that upregulation of PIWI proteins were negatively correlated with patient survival whereas downregulation of PIWI proteins reduced the number of G2/M phase cells and enhanced expression of p53 protein, thus inhibiting proliferation and promoting apoptosis. Furthermore, PIWI proteins emerging expression could increase resistance to cisplatin [50]. In IBCs with normal expression or aberrant expression of PIWI proteins, synthetic piRNAs targeting *PIWI* genes could be potential tools in transcriptional silencing and antibodies targeting PIWI proteins could also constitute another pertinent approach to antagonize cancer cells proliferation at post-transcriptional level [50]. Concerning PIWIL2 and PIWIL4 proteins status in our series, we observed that IBCs with PIWIL2–PIWIL4 underexpression where characterized by downregulation of molecules involved in chromatin accessibility, genome methylation and increased inflammatory/cytotoxic immune reaction. Recent in silico studies suggested that varieties of reexpressed TEs were associated with activation of innate immune genes and could enhance production of polypeptides that are presented by cancer cells on MHC I molecules and subject to surveillance by the adaptive immune system [61]. These findings suggest that TEs reexpression whether spontaneous (in IBCs with PIWIL2–PIWIL4 downregulation) or induced under epigenetic therapy using hypomethylating agents has important potential clinical implications for cancer immunotherapy [53,61,63]. Therapeutic reactivation of tumor-specific TEs may synergize with immunotherapy by inducing both inflammation and the display of potentially immunogenic neoantigens. Thus, IBCs with PIWIL2–PIWIL4 downregulation could benefit from immunotherapy by using checkpoints inhibitors because of an important cytotoxic immune reaction and reexpression of many neoantigens and TEs by cancer cells. Conversely, IBCs with functional PIWI–piRNA pathway characterized by PIWIL2–PIWIL4 normal level of expression could be therapeutically reprogrammed in IBCs with inactivated PIWI–piRNA pathway by combining hypomethylating agents (such as DNMT1 repressors) and inhibitors of checkpoints [59]. Because hypomethylating agents induce PD-1 promoter demethylation, overexpression of PD-1 on immune cells and T-cell exhaustion, combining hypomethylating drugs with anti PD-1 checkpoint therapy could optimize the beneficial effects of epigenetic treatments [64].

## 5. Conclusions

For the first time to our knowledge, we identified a generic expression pattern of inactivation of the PIWI–piRNA pathway present in 17 different malignant tumors, including IBCs, characterized by underexpression of the pi-RISCs catalytic and oncosuppressive effector proteins PIWIL2 and PIWIL4 and aberrant emergence of PIWIL1 and PIWIL3. Identification and characterization of the PIWI–piRNA pathway in IBCs open interesting therapeutic perspectives such as piRNAs, combination of hypomethylating drugs with checkpoints immunotherapies and anti-PIWIL1/PIWIL3 antibodies.

## Figures and Tables

**Table 1:** PIWIL1-2-3-4 mRNA expression levels in a panel of normal tissues and corresponding malignant tumors.

	Normal Tissue RNA Level					Malignant Tumors RNA Level				
Tissue	Number	PIWIL1	PIWIL2	PIWIL3	PIWIL4	Number	PIWIL1	PIWIL2	PIWIL3	PIWIL4
Anal canal	17	0 (0–9.33)	17.9 (4.77–64.0)	0 (0–1.44)	319 (30.0–426)	48	0.92 (0–96.2)	7.82 (1.25–717)	0 (0–0)	48.6 (10.8–390)
Head and neck	27	0 (0–2.42)	51.2 (1.98–160)	0 (0–0.54)	68.8 (35.7–251)	50	0.50 (0–187)	12.2 (0.37–409)	0 (0–0)	42.4 (7.45–282)
Prostate	8	0 (0–2.25)	23.0 (8.98–60.7)	0 (0–0)	72.7 (34.2–124)	48	0 (0–107)	13.5 (1.82–47.2)	0 (0–1.55)	40.8 (14.9–84.8)
Ovary	27	1.32 (0–36.5)	54.5 (12.9–119)	0 (0–0.94)	160 (84.3–277)	52	11.9 (0–229.6)	10.6 (0.96–148)	0 (0–23.7)	100 (13.2–1442)
Thyroid	9	0.50 (0–6.17)	13.7 (8.05–146)	0 (0–0)	57.9 (40.4–95.6)	31	8.93 (0–455)	13.3 (2.41–40.3)	0 (0–0.51)	48.0 (13.1–130)
Cervix	14	0 (0–2.98)	47.2 (9.12–108)	0 (0–0)	124 (36.9–332)	37	2.14 (0–785)	7.94 (1.14–549)	0 (0–0)	68.9 (7.56–825)
Skin	9	0 (0–14.0)	40.7 (22.1–110)	0 (0–0)	67.5 (25.7–134)	27	0 (0–5.64)	11.6 (0–58.2)	0 (0–49.1)	47.9 (0–223)
Endometrium	8	0.29 (0–5.11)	69.9 (47.1–99.8)	0 (0–0)	177 (111–232)	29	5.87 (0–478)	7.57 (1.24–59.5)	0 (0–5.29)	46.5 (9.39–222)
Colon	30	1.37 (0–76.4)	14.0 (6.24–57.9)	0 (0–0)	313 (98.3–467)	49	20.4 (0–1569)	7.28 (1.37–36.1)	0 (0–1.29)	250 (32.0–984)
Lung	16	0 (0–2.24)	9.61 (1.54–32.0)	0 (0–0)	90.5 (55.3–151)	54	0 (0–428)	6.85 (0–236)	0 (0–89.0)	73.6 (0–360)
Kidney	18	7.24 (0–24.8)	20.5 (4.30–99.4)	0 (0–0)	97.5 (53.3–182)	22	3.44 (0–20.2)	16.5 (1.79–72.0)	0 (0–0)	118 (25.4–753)
Pancreas	10	0 (0–5.22)	24.4 (17.6–70.2)	0 (0–0)	530 (380–831)	22	6.34 (0–166)	37.1 (5.79–80.0)	0 (0–0)	414 (118–1536)
Liver	9	0 (0–3.92)	14.2 (7.01–36.3)	0 (0–0)	65.2 (25.4–252)	31	0 (0–6.59)	8.36 (0–102)	0 (0–17.5)	33.9 (5.46–354)
Bladder	14	0 (0–16.9)	49.7 (0–129)	0 (0–0)	121 (80.7–151)	49	0 (0–47.7)	4.65 (0–604)	0 (0–19.3)	25.0 (2.05–532)
Stomach	11	0 (0–174)	10.1 (0–45.7)	0 (0–0)	156 (116–252)	29	4.44 (0–961)	8.28 (1.35–62.1)	0 (0–1.77)	241 (107–522)
Brain	19	1.44 (0–18.8)	26.4 (7.08–67.6)	0 (0–0)	103 (46.5–192)	50	0 (0–3.14)	29.3 (3.84–356)	0 (0–0.34)	56.2 (3.39–176)
Breast	11	0 (0–7.87)	66.8 (28.5–118)	0 (0–0)	138 (60.2–190)	96	0 (0–6.03)	11.2 (0–758)	0 (0–39.2)	34.9 (7.43–176)

mRNA levels in the samples normalized to obtain a basal mRNA level (smallest amount of mRNA quantifiable, i.e., Ct = 35) equal to 1.

**Table 2:** PIWIL1-2-3-4 proteins histological score in a multitumor panel (*n* = 17) of normal tissues and malignant tumors.

Normal Tissues Protein Level	PIWIL1	PIWIL2	PIWIL3	PIWIL4	Malignant Tumors Protein Level	PIWIL1	PIWIL2	PIWIL3	PIWIL4
Testis (*n* = 10)	2	2	1.5	2	Testis (*n* = 10)	1	0–1	0	0–1
Anal canal (*n* = 10)	0	1	0	1	Anal canal (*n* = 10)	1	0–1	0	0–1
Head and neck (*n* = 10)	0	1	0	1	Head and neck (*n* = 10)	0	0–1	0	0–1
Prostate (*n* = 10)	0	1	0	1	Prostate (*n* = 10)	0	0–1	0	0–1
Ovary (*n* = 10)	1	1	0	1	Ovary (*n* = 10)	1–2	0–1	0	0–1
Thyroid (*n* = 10)	1	1	0	1	Thyroid (*n* =10)	1–2	0–1	0	0–1
Cervix (*n* = 10)	0	1	0	1	Cervix (*n* = 10)	0–1	0–1	0	0–1
Skin (*n* = 10)	0	1	0	1	Skin (*n* = 10)	0	0–1	0	0–1
Endometrium (*n* = 10)	1	1	0	1	Endometrium (*n* = 10)	1–2	0–1	0	0–1
Colon (*n* = 10)	1	1	0	1	Colon (*n* = 10)	1–2	0–1	0	0–1
Lung (*n* = 10)	0	1	0	1	Lung (*n* = 10)	0	0–1	0	0–1
Kidney (*n* = 10)	1	1	0	1	Kidney (*n* = 10)	1–2	0–1	0	0–1
Pancreas (*n* = 10)	0	1	0	1	Pancreas (*n* = 10)	1–2	0–1	0	0–1
Liver (*n* = 10)	0	1	0	1	Liver (*n* = 10)	0	0–1	0	0–1
Bladder (*n* = 10)	0	1	0	1	Bladder (*n* = 10)	0	0–1	0	0–1
Stomach (*n* = 10)	0	1	0	1	Stomach (*n* = 10)	0–1	0–1	0	0–1
Brain (*n* = 10)	1	1	0	1	Brain (*n* = 10)	0–1	0–1	0	0–1

Comparison of PIWIL1-2-3-4 expression levels in germinal and somatic tissues aimed to specify their pure germinal or mixed germinal and somatic status. PIWIL3 gene was only expressed in normal germ cells and not in normal somatic cells (pure germinal status). PIWIL2 and PIWIL4 genes were expressed in both normal germinal and somatic tissues (mixed germinal and somatic status). PIWIL1 gene had two statuses according to tissues origin: a pure germinal status in head and neck, prostate, bladder, liver, pancreas, lung, cervix, stomach and anal canal normal tissues and a mixed germinal and somatic status in kidney, brain, endometrium, ovary, colon and thyroid normal tissues.

**Table 3:** PIWIL1-2-3-4 mRNA levels of expression in a series of IBCs (*n* = 526) with molecular subtypes.

RNA Level	PIWIL1	PIWIL2	PIWIL3	PIWIL4
Normal Tissues (*n* = 14)	0	1 (0.27–2.52)	0	1 (0.39–3.10)
IBCs (*n* = 526)	0 (0–15.53) N: 367 (69.8%) A: 159 (30.2%)	0.34 (0–19.38) U: 254 (48.3%) N: 272 (51.7%)	0 (0–151.97) N: 495 (94.1%) A: 31 (5.9%)	0.37 (0–2.96) U: 228 (43.3%) N: 298 (56.7%)
NNN (*n* = 101)	0 (0–15.53) N: 76 (75.2%) A: 25 (24.8%)	0.32 (0–16) U: 53 (52.5%) N: 48 (47.5%)	0 (0–15.97) N: 98 (97%) A: 3 (3%)	0.61 (0.05–2.96) U: 29 (28.7%) N: 72 (71.3%)
RH-HER2+ (*n* = 73)	0 (0–4.08) N: 51 (69.9%) A: 22 (30.1%)	0.31 (0–4.35) U: 38 (52.1%) N: 35 (47.9%)	0 (0–53.36) N: 62 (84.9%) A: 11 (15.1%)	0.37 (0–1.75) U: 29 (39.7%) N: 44 (60.3%)
RH+HER2- (*n* = 294)	0 (0–14.45) N: 203 (69.0%) A: 91 (31.0%)	0.33 (0–18.39) U: 148 (50.3%) N: 146 (49.7%)	0 (0–26.49) N: 282 (95.9%) A: 12 (4.1%)	0.35 (0–2.82) U: 139 (47.3%) N: 155 (52.7%)
RH+HER2+ (*n* = 58)	0 (0–2.96) N: 37 (63.8%) A: 21 (36.2%)	0.59 (0.07–19.38) U: 15 (25.9%) N: 43 (74.1%)	0 (0–9.21) N: 53 (91.4%) A: 5 (8.6%)	0.32 (0.06–1.45) U: 31 (53.4%) N: 27 (46.6%)

U—underexpression; O—overexpression; N—normal expression; A—aberrant emerging expression.

## Supplementary Materials

The following are available online at https://www.mdpi.com/2072-6694/12/10/2833/s1, Figure S1: PIWI-piRNA pathway biogenesis and function in maintaining integrity of the genome, Figure S2: ×400 and ×1000; PIWIL1-2-3-4 proteins immunostaining in normal testis and breast tissues, Figure S3: ×10 and ×200; Early progressive PIWIL2 and PIWIL4 downregulation in pre-invasive breast lesions such as atypical ductal hyperplasia (ADH) and ductal carcinoma in situ (DCIS), Figure S4: Statistically significant correlation between PIWIL2 and PIWIL4 mRNA expression levels *p* < 0.0001 in the series of 526 IBCs, Figure S5, a: MFS curves of patient groups according to PIWIL1-4 mRNA expression levels in the series of 526 IBCs; b: OS curves of patient groups according to PIWIL1-4 mRNA expression levels in the series of 526 IBCs, Figure S6, a: PIWIL1-2-3-4 mRNA expression levels in a panel of normal tissues; b: PIWIL1-2-3-4 mRNA expression levels in a panel of malignant tumors, Table S1: Characteristics of the 526 breast tumors, Table S2: Number of normal and tumoral samples for each tissue type, Table S3: Sequences of primers used for RT-qPCR, Table S4: Statistical analysis of mRNA expression levels of PIWIL2 and PIWIL4 relative to respective protein expression levels in a series of 62 breast tumors, Table S5: Correlation between PIWIL1, PIWIL2, PIWIL3 and PIWIL4 mRNA expression levels with classical clinicopathological factors in IBCs, Table S6: PIWIL1-2-3-4 protein levels of expression in a series of IBCs (n = 150) with molecular subtypes, Table S7: Percentage of PIWIL1-3 RNA expression and PIWIL2-4 RNA underexpression in a multitumor panel, Table S8: Piwil2-4 genes are associated with hallmarks of cancer.
