# Supplementary material for: Biopathological Significance of PIWI–piRNA Pathway Deregulation in Invasive Breast Carcinomas
Source: Cancers (Basel). 2020 Sep 30;12(10):2833. doi: 10.3390/cancers12102833 (PMC7600338; doi:10.3390/cancers12102833)
Supplement: Supplementary file 1 [file cancers-12-02833-s001.pdf]

cancers

Supplementary Material: Biopathological Significance of PIWI–piRNA Pathway Deregulation in Invasive Breast Carcinomas

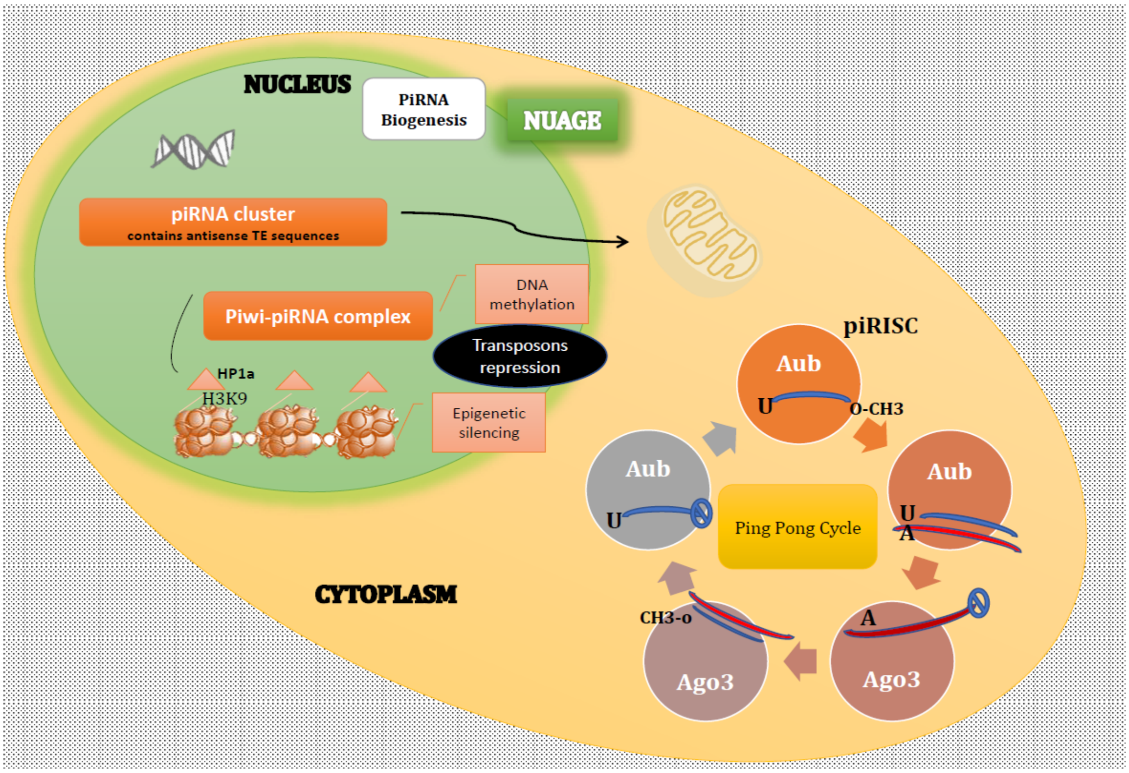

Figure S1. PIWI-piRNA pathway biogenesis and function in maintaining integrity of the genome.

A

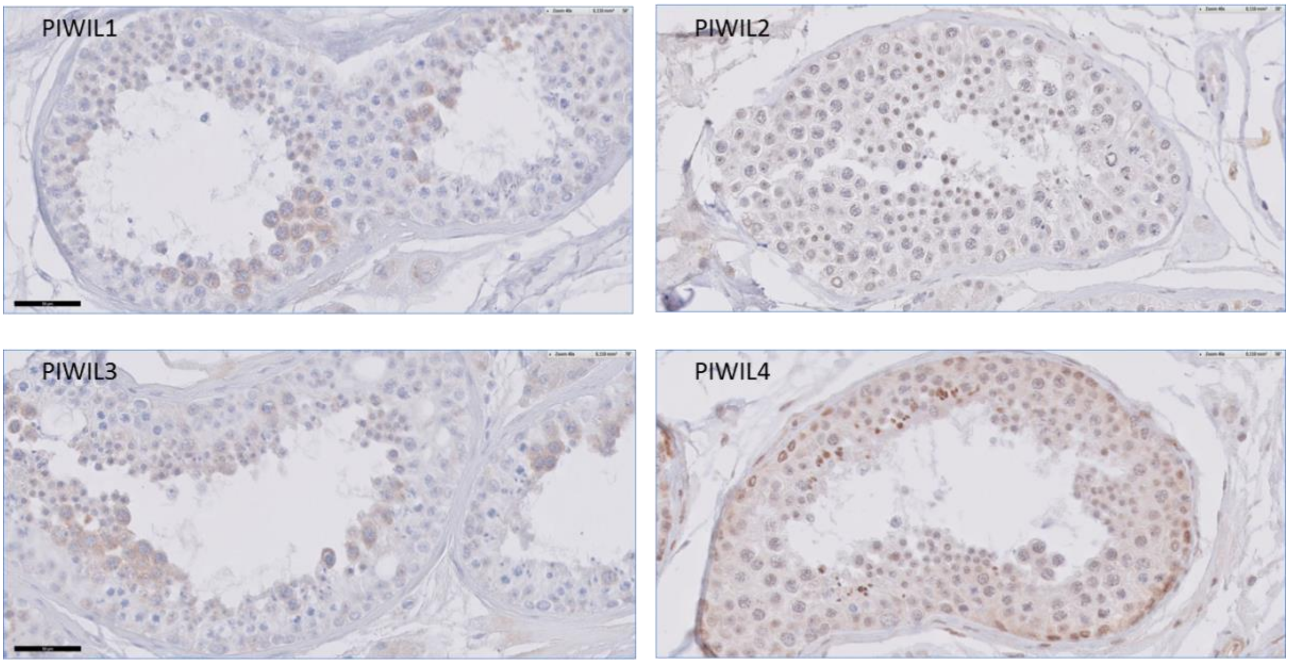

In normal testis, all 4 PIWIL proteins are expressed

B

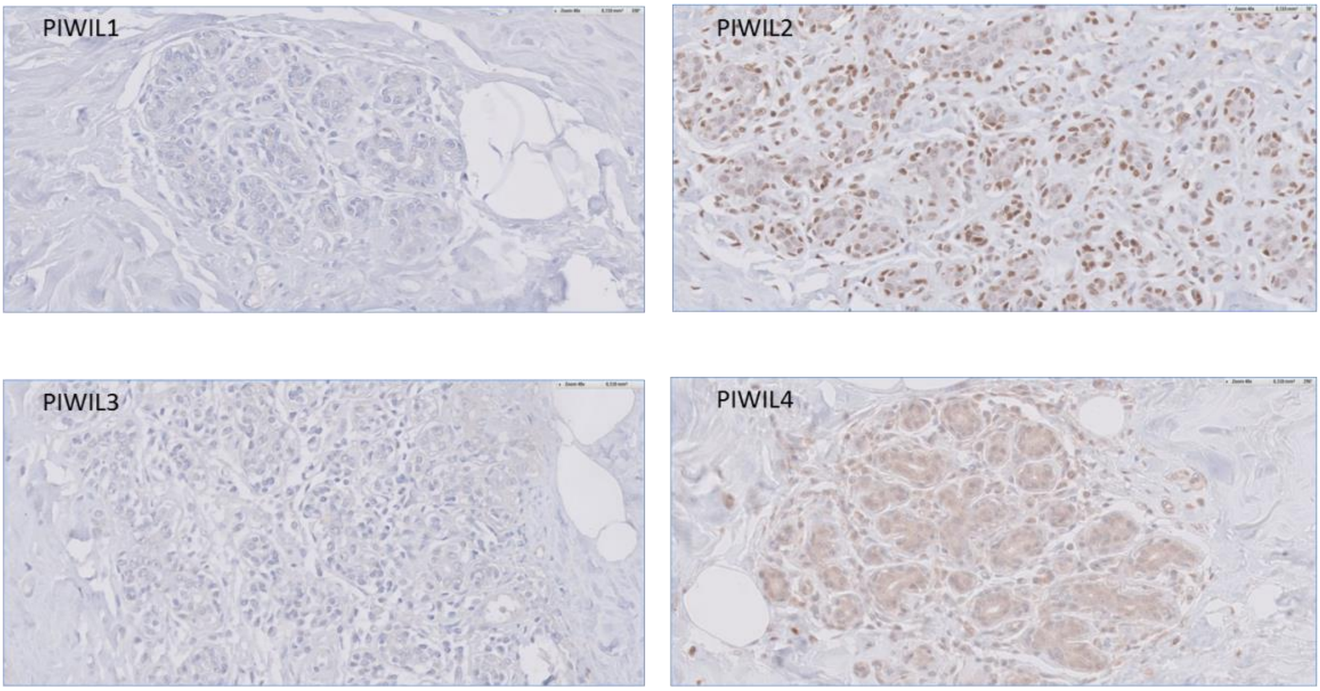

In normal breast, only PIWIL2 and PIWIL4 proteins are expressed

Figure S2. PIWIL1-2-3-4 proteins immunostaining in normal testis and breast tissues

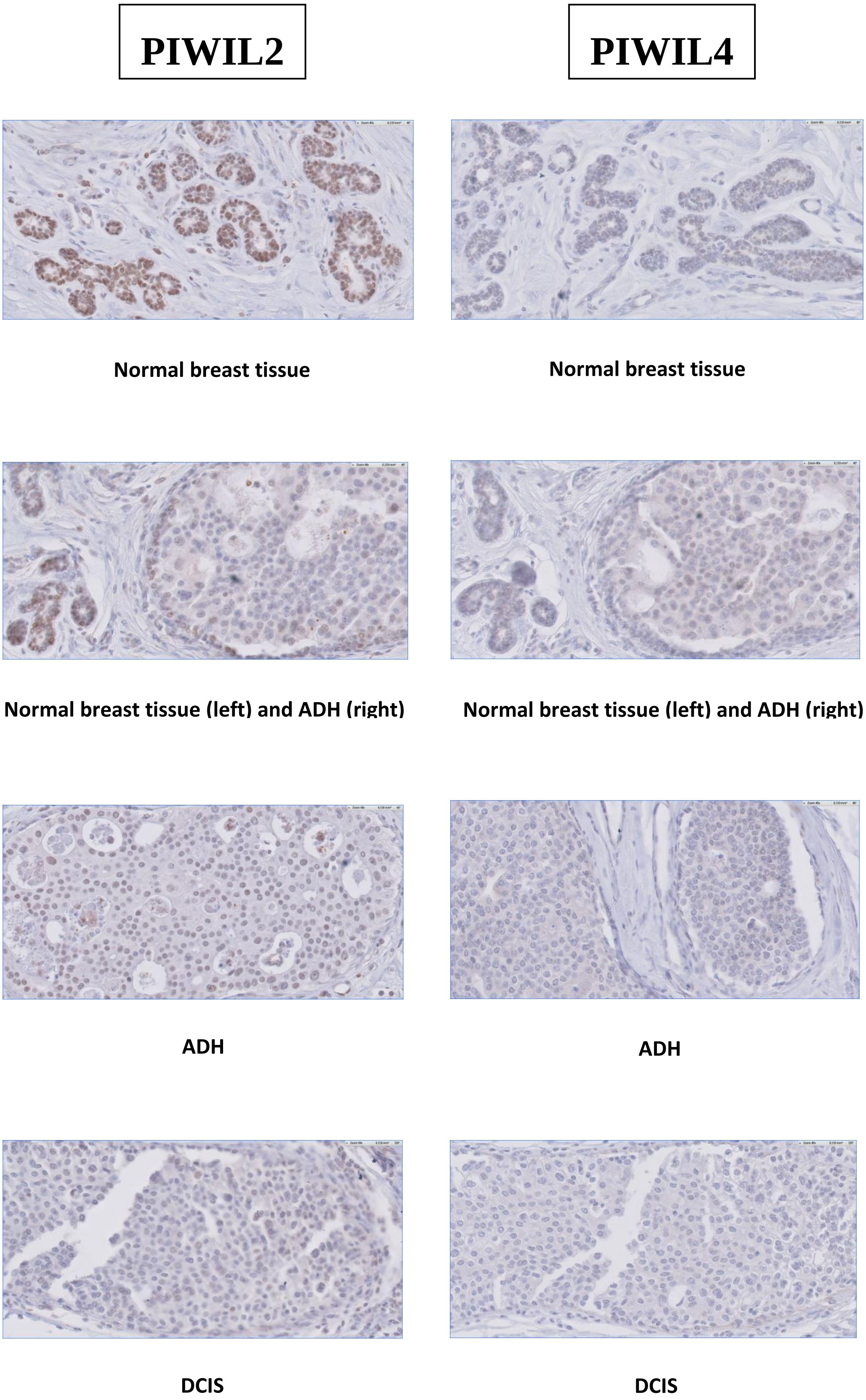

Figure S3. Early progressive PIWIL2 and PIWIL4 downregulation in pre-invasive breast lesions such as atypical ductal hyperplasia (ADH) and ductal carcinoma in situ (DCIS).

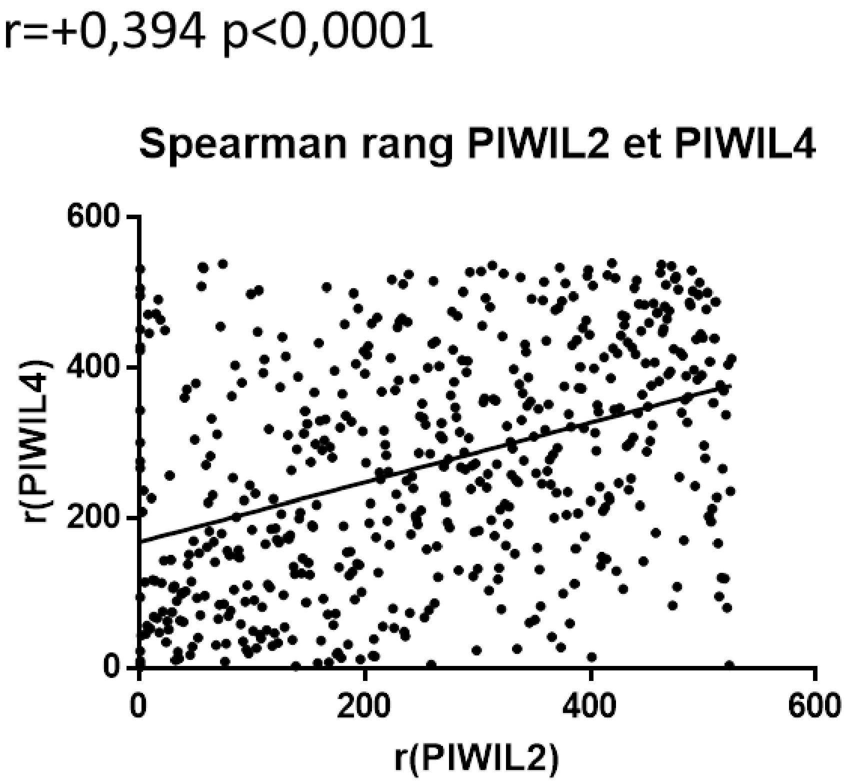

Figure S4. Statistically significant correlation between PIWIL2 and PIWIL4 mRNA expression levels [$p < 0.0001$] in the series of 526 IBCs.

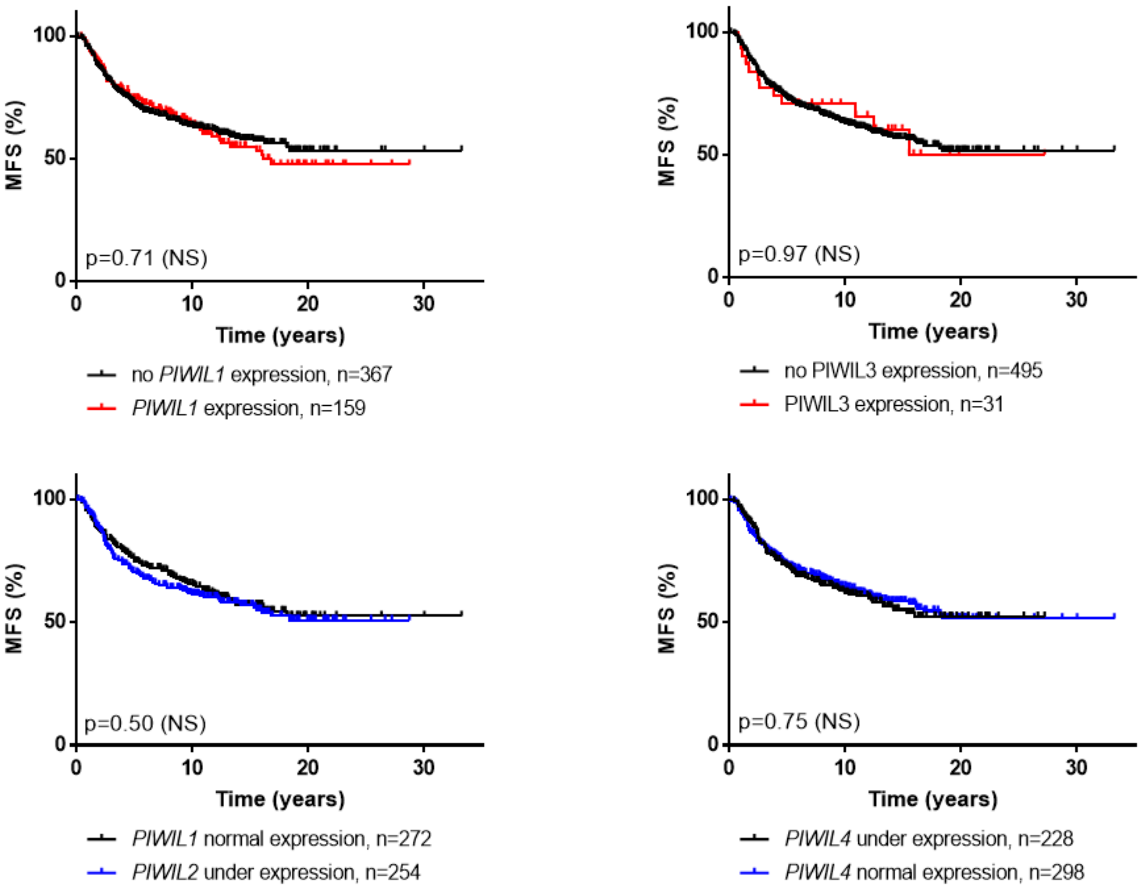

Figure S5. MFS curves of patient groups according to *PIWIL1-4* mRNA expression levels in the series of 526 IBCs.

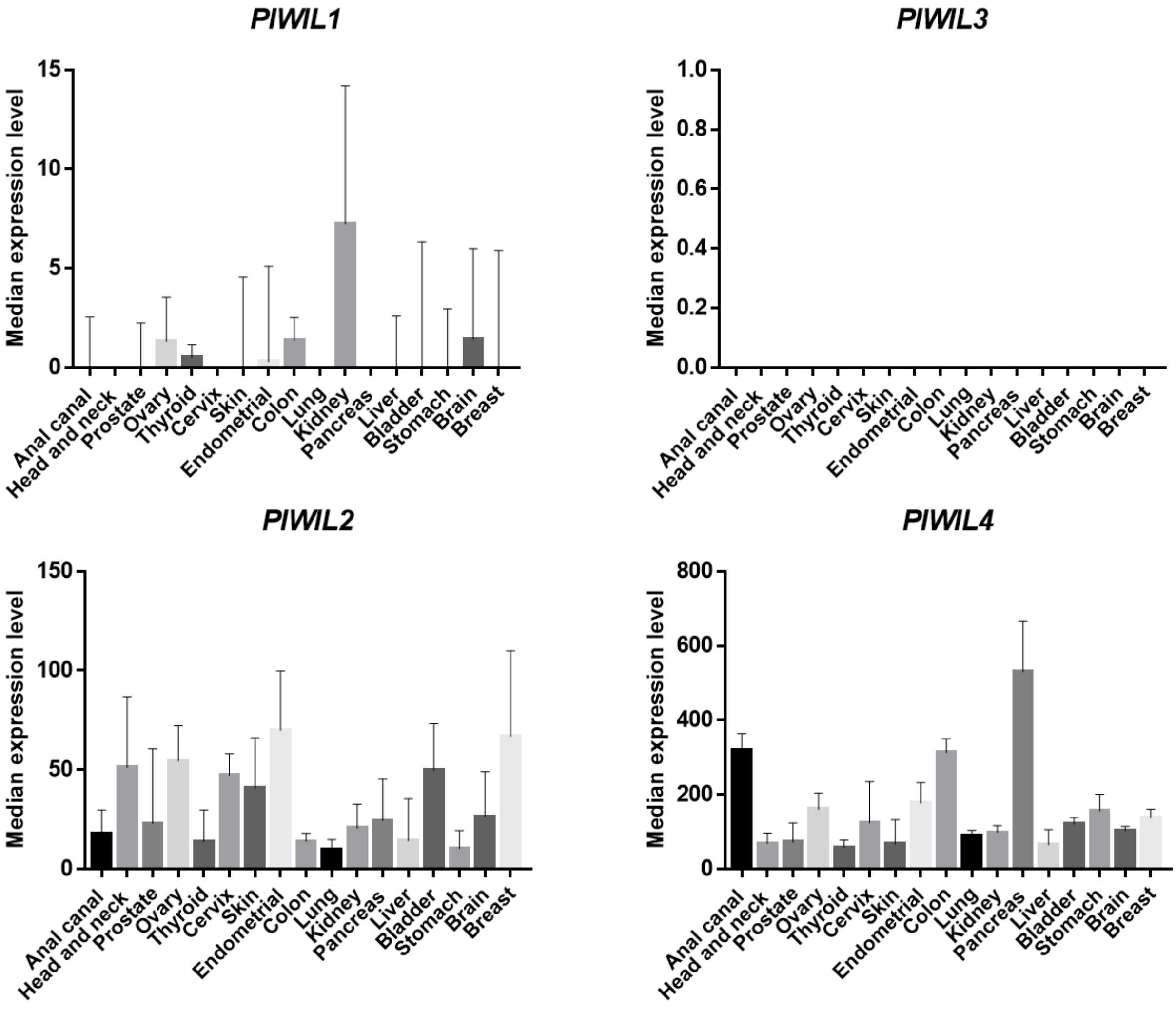

Figure S6a. *PIWIL1-2-3-4* mRNA expression levels in a panel of normal tissues

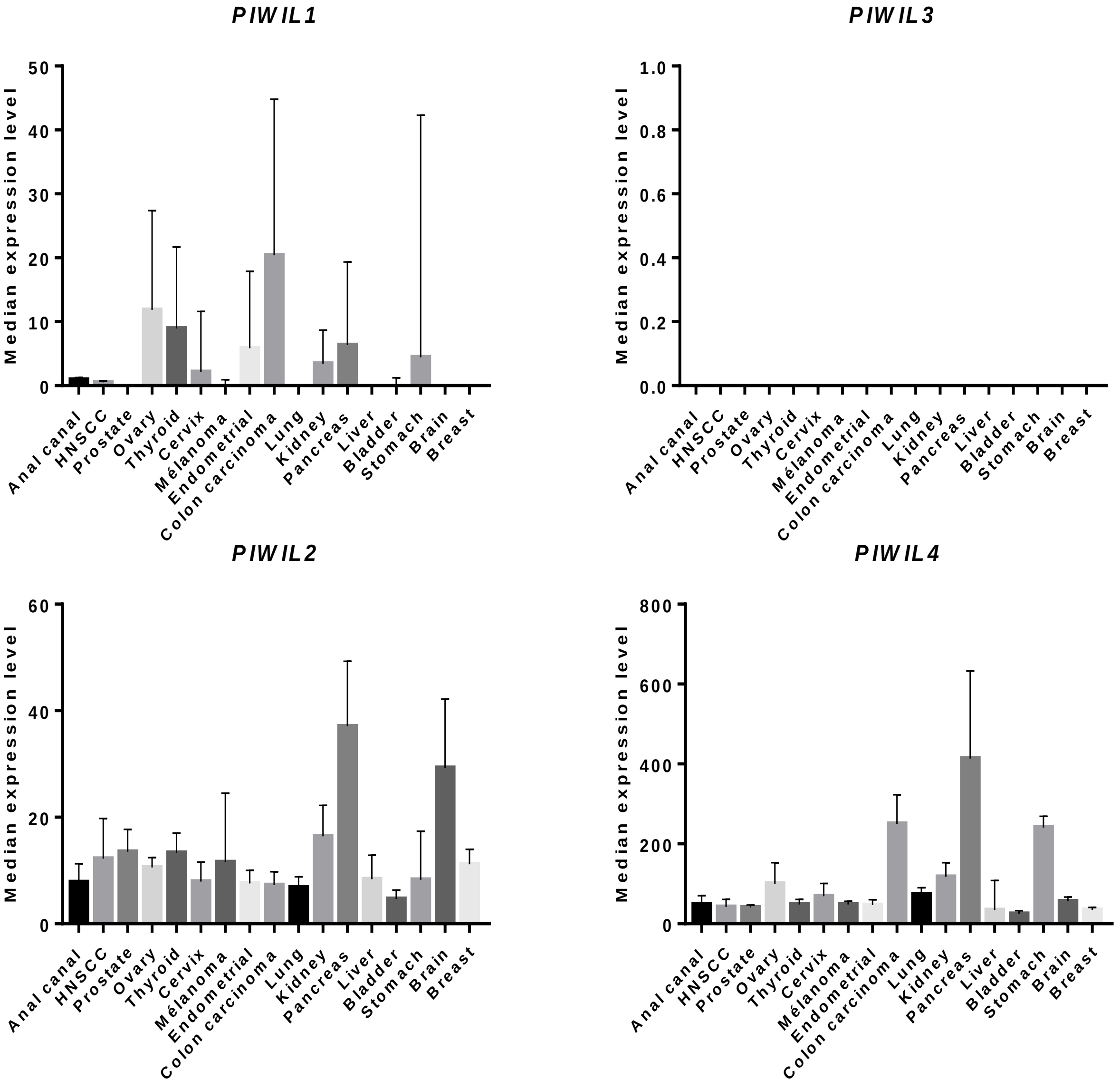

Figure 6b. *PIWIL1-2-3-4* mRNA expression levels in a panel of malignant tumors

Table S1. Characteristics of the 526 breast tumors.

	Number of patients (%)	Number with metastases (%)	p-value[a]
Total	526 (100)	209 (39.7)	
Age			
≤50	125 (23.8)	52 (41.6)	0.51 (NS)
>50	401 (76.2)	157 (39.2)	

SBR histological grade [b,c]			
I	60 (11.7)	12 (20.0)	
II	241 (47.2)	100 (41.5)	**0.0018**
III	210 (41.1)	93 (44.3)	
Lymph node status [d]			
0	160 (30.7)	48 (30.0)	
1-3	248 (47.6)	87 (35.1)	**< 0.0001**
>3	113 (21.7)	72 (63.7)	
Macroscopic tumor size e			
≤25mm	248 (48.1)	76 (30.6)	**< 0.0001**
>25mm	268 (51.9)	132 (49.3)	
ERα status			
Negative	181 (34.4)	76 (42.0)	0.096 (NS)
Positive	345 (65.6)	133 (38.6)	
PR status			
Negative	254 (48.3)	109 (42.9)	**0.028**
Positive	272 (51.7)	100 (36.8)	
ERBB2 status			
Negative	395 (75.1)	152 (38.5)	0.19 (NS)
Positive	131 (24.9)	57 (43.5)	
Molecular subtypes			
HR- ERBB2-	101 (19.2)	38 (37.6)	
HR- ERBB2+	73 (13.9)	36 (49.3)	0.072 (NS)
HR+ ERBB2-	294 (55.9)	114 (38.8)	
HR+ ERBB2+	58 (11.0)	21 (36.2)	
PIK3CA mutation status f			
wild type	354 (68.1)	145 (41.0)	0.21 (NS)
mutated	166 (31.9)	61 (36.7)	

[a] Log-rank test. NS: not significant [b] Scarff Bloom Richardson classification [c] Information available for 511 patients [d] Information available for 521 patients [e] Information available for 516 patients [f] Information available for 520 patients.

Table S2. Number of normal and tumoral samples for each tissue type.

Tissue type	Number of normal tissues	Number of tumoral tissues	Histological types of tumoral tissues	Number of tumor subtypes
Anal canal	17	48	Epidermoïd carcinomas	
Head and neck	27	50	Epidermoïd carcinomas	
Prostate	7	48	Adenocarcinomas	
Ovary	27	52	Serous carcinomas	
Thyroid	9	31	Papillary carcinomas	
Cervix	14	37	Epidermoïd carcinomas	
Skin	9	27	Epidermoïd carcinomas	
Endometrium	8	29	Adenocarcinomas	
Colon	30	49	colon metastatic carcinomas	25
Colon			locally advanced colon carcinomas	24
Lung	16	54	adenocarcinomas	38

Lung			squamous cell carcinomas	16
Kidney	18	22	Clear cells carcinomas	
Pancreas	11	22	Adenocarcinomas	
Liver	10	31	Hepatocellular carcinomas	
Bladder	14	49	superficial urothelial carcinoma	25
Bladder			invasive urothelial carcinoma	24
Stomach	11	29	Adenocarcinomas	
Brain	21	50	low grade gliomas (grade II)	25
			glioblastomas (grade IV)	25

Table S3. Sequences of primers used for RT-qPCR.

Gene	Upper primer (5′ to 3′)	Lower primer (5′ to 3′)	PCR product size (pb)
**PIWIL1**	GTGGGCTCCACTGCCAGTCA	CTGTTCCTCCTGCTGTTCCTCTCT	115
**PIWIL2**	CTGGGCATTGAAACAGTTTCTAAGAC	TCGACCTAAAATGCCTCTACCTGAT	85
**PIWIL3**	GCACCTGGATCAGCTACAACCCA	TCTTGGCTGCAGAGGTCTAACCACT	99
**PIWIL4**	CTTGTGGACAACATCCAGAGGAATAC	CACAATCCGGCCAGTCAGAGA	99
**PIWIL2-V5**	CCTATCCCTTTCATACTGTTCTGGAT	CCTGGAAGTGTTCTTTATTCTGCTG	103
**TBP**	TGCACAGGAGCCAAGAGTGAA	CACATCACAGCTCCCCACCA	132

Table S4. Statistical analysis of mRNA expression levels of *PIWIL2* and *PIWIL4* relative to respective protein expression levels in a series of 62 breast tumors.

	**mRNA expression level**		
**Gene**	**under-expression protein group**	**normal expression protein group**	***p*-value [a]**
	n = 37	*n* = 25	
PIWIL2	0.26 (0-29.1)[b]	0.45 (0.05-1.43)	**0.0019**
	n = 23	*n* = 39	
PIWIL4	0.33 (0.05-0.73)	0.79 (0.18-3.62)	**< 0.0001**

[a] Mann Withney Test; [b] Median (range) of gene mRNA expression level.

Table S5. Correlation between *PIWIL1, PIWIL2, PIWIL3* and *PIWIL4* mRNA expression levels with classical clinicopathological factors in IBCs.

	Total population	%	Number of patients (%) *PIWIL4* mRNA underexpression (≤0,33 relative to normals)	%	*PIWIL4* mRNA normal expression	%	p-value a
Total	526	100%	228	43,3%	298	56,7%	
Age							
≤50	125	23,8%	50	40,0%	75	60,0%	0,39 (NS)
>50	401	76,2%	178	44,4%	223	55,6%	
SBR histological grade b, c							
I	60	11,7%	23	38,3%	37	61,7%	0,50 (NS)
II	241	47,2%	105	43,6%	136	56,4%	
III	210	41,1%	98	46,7%	112	53,3%	
Lymph node status d							
0	160	30,7%	70	43,8%	90	56,3%	0,71 (NS)
1-3	248	47,6%	110	44,4%	138	55,6%	
>3	113	21,7%	45	39,8%	68	60,2%	
Macroscopic tumor size e							
≤25mm	248	48,1%	103	41,5%	145	58,5%	0,46 (NS)
>25mm	268	51,9%	120	44,8%	148	55,2%	
ERα status							
Negative	181	34,4%	58	32,0%	123	68,0%	**0,0002**
Positive	345	65,6%	170	49,3%	175	50,7%	
PR status							
Negative	254	48,3%	101	39,8%	153	60,2%	0,11 (NS)
Positive	272	51,7%	127	46,7%	145	53,3%	
ERBB2 status							
Negative	395	75,1%	168	42,5%	227	57,5%	0,51 (NS)
Positive	131	24,9%	60	45,8%	71	54,2%	
Molecular subtypes							
HR- ERBB2-	101	19,2%	29	28,7%	72	71,3%	**0,0037**
HR- ERBB2+	73	13,9%	29	39,7%	44	60,3%	
HR+ ERBB2-	294	55,9%	139	47,3%	155	52,7%	
HR+ ERBB2+	58	11,0%	31	53,4%	27	46,6%	
PIK3CA mutation status f							
wild type	354	68,1%	152	42,9%	202	57,1%	0,63 (NS)
mutated	166	31,9%	75	45,2%	91	54,8%	
MKI67 mRNA expression median	8,55 (0,22-81,5)		8,97 (0,28-72,0)		8,08 (0,22-81,5)		0,11 (NS) h
Metastasis							
No	317	60,3%	136	42,9%	181	57,1%	0,80 (NS)
Yes	209	39,7%	92	44,0%	117	56,0%	

NS: not significant
a Chi-squared Test
b Scarff Bloom Richardson classification
c Information available for 511 patients
d Information available for 521 patients
e Information available for 516 patients
f Information available for 520 patients
h Mann-Whitney Test

	Number of patients (%)						
	Total population	%	*PIWIL2* mRNA underexpression (≤0,33 relative to normals)	%	*PIWIL2* mRNA normal expression	%	p-value a
Total	526	100%	254	48,3%	272	51,7%	
Age							
≤50	125	23,8%	55	44,0%	70	56,0%	0,27 (NS)
>50	401	76,2%	199	49,6%	202	50,4%	
SBR histological grade b, c							
I	60	11,7%	25	41,7%	35	58,3%	0,10 (NS)
II	241	47,2%	109	45,2%	132	54,8%	
III	210	41,1%	113	53,8%	97	46,2%	
Lymph node status d							
0	160	30,7%	81	50,6%	79	49,4%	0,62 (NS)
1-3	248	47,6%	114	46,0%	134	54,0%	
>3	113	21,7%	56	49,6%	57	50,4%	
Macroscopic tumor size e							
≤25mm	248	48,1%	114	46,0%	134	54,0%	0,31 (NS)
>25mm	268	51,9%	135	50,4%	133	49,6%	
ERα status							
Negative	181	34,4%	92	50,8%	89	49,2%	0,40 (NS)
Positive	345	65,6%	162	47,0%	183	53,0%	
PR status							
Negative	254	48,3%	140	55,1%	114	44,9%	**0,0025**
Positive	272	51,7%	114	41,9%	158	58,1%	
ERBB2 status							
Negative	395	75,1%	201	50,9%	194	49,1%	**0,039**
Positive	131	24,9%	53	40,5%	78	59,5%	
Molecular subtypes							
HR- ERBB2-	101	19,2%	53	52,5%	48	47,5%	**0,004**
HR- ERBB2+	73	13,9%	38	52,1%	35	47,9%	
HR+ ERBB2-	294	55,9%	148	50,3%	146	49,7%	
HR+ ERBB2+	58	11,0%	15	25,9%	43	74,1%	
PIK3CA mutation status f							
wild type	354	68,1%	182	51,4%	172	48,6%	0,087 (NS)
mutated	166	31,9%	72	43,4%	94	56,6%	
MKI67 mRNA expression median	8,55 (0,22-81,5)		8,96 (0,28-79,8)		8,12 (0,22-81,5)		0,29 (NS) h
Metastasis							
No	317	60,3%	149	47,0%	168	53,0%	0,47 (NS)
Yes	209	39,7%	105	50,2%	104	49,8%	

NS: not significant
a Chi-squared Test
b Scarff Bloom Richardson classification
c Information available for 511 patients
d Information available for 521 patients
e Information available for 516 patients
f Information available for 520 patients
h Mann-Whitney Test

Supplemental Table 4: Relationships between *PIWIL3* transcript levels and classical clinical biological parameters in a series of 526 breast cancer

			Number of patients (%)				
	Total population	%	*PIWIL3* mRNA normal expression	%	*PIWIL3* mRNA emergence of expression (Ct<38)	%	p-value [a]
Total	526	100%	495	94.1%	31	5.9%	
Age							
≤50	125	23.8%	122	97.6%	3	2.4%	0.058 (NS)
>50	401	76.2%	373	93.0%	28	7.0%	
SBR histological grade [b, c]							
I	60	11.7%	58	96.7%	2	3.3%	0.54 (NS)
II	241	47.2%	227	94.2%	14	5.8%	
III	210	41.1%	195	92.9%	15	7.1%	
Lymph node status [d]							
0	160	30.7%	147	91.9%	13	8.1%	0.36 (NS)
1-3	248	47.6%	235	94.8%	13	5.2%	
>3	113	21.7%	108	95.6%	5	4.4%	
Macroscopic tumor size [e]							
≤25mm	248	48.1%	231	93.1%	17	6.9%	0.44 (NS)
>25mm	268	51.9%	254	94.8%	14	5.2%	
ERα status							
Negative	181	34.4%	166	91.7%	15	8.3%	0.091 (NS)
Positive	345	65.6%	329	95.4%	16	4.6%	
PR status							
Negative	254	48.3%	233	91.7%	21	8.3%	**0.026**
Positive	272	51.7%	262	96.3%	10	3.7%	
ERBB2 status							
Negative	395	75.1%	380	96.2%	15	3.8%	**0.0004**
Positive	131	24.9%	115	87.8%	16	12.2%	
Molecular subtypes							
HR- ERBB2-	101	19.2%	98	97.0%	3	3.0%	**0.0017**
HR- ERBB2+	73	13.9%	62	84.9%	11	15.1%	
HR+ ERBB2-	294	55.9%	282	95.9%	12	4.1%	
HR+ ERBB2+	58	11.0%	53	91.4%	5	8.6%	
PIK3CA mutation status [f]							
wild type	354	68.1%	335	94.6%	19	5.4%	0.40 (NS)
mutated	166	31.9%	154	92.8%	12	7.2%	
MKI67 mRNA expression							
median	8.55 (0.22-81.5)		8.30 (0.22-81.5)		10.5 (2.09-79.8)		0.12 (NS) [h]
Metastasis							
No	317	60.3%	298	94.0%	19	6.0%	0.90 (NS)
Yes	209	39.7%	197	94.3%	12	5.7%	

NS: not significant
[a] Chi-squared Test
[b] Scarff Bloom Richardson classification
[c] Information available for 511 patients
[d] Information available for 521 patients
[e] Information available for 516 patients
[f] Information available for 520 patients
[h] Mann-Whitney Test

cancers

Supplemental Table 4: Relationships between *PIWIL1* transcript levels and classical clinical biological parameters in a series of 526 breast cancer

			Number of patients (%)				
	Total population	%	*PIWIL1* mRNA normal expression (Ct>38)	%	*PIWIL1* mRNA emergence of expression (Ct<38)	%	p-value [a]
Total	526	100%	367	69.8%	159	30.2%	
Age							
≤50	125	23.8%	93	74.4%	32	25.6%	0.20 (NS)
>50	401	76.2%	274	68.3%	127	31.7%	
SBR histological grade [b, c]							
I	60	11.7%	38	63.3%	22	36.7%	0.48 (NS)
II	241	47.2%	172	71.4%	69	28.6%	
III	210	41.1%	146	69.5%	64	30.5%	
Lymph node status [d]							
0	160	30.7%	97	60.6%	63	39.4%	**0.0065**
1-3	248	47.6%	180	72.6%	68	27.4%	
>3	113	21.7%	87	77.0%	26	23.0%	
Macroscopic tumor size [e]							
≤25mm	248	48.1%	161	64.9%	87	35.1%	**0.021**
>25mm	268	51.9%	199	74.3%	69	25.7%	
ERα status							
Negative	181	34.4%	129	71.3%	52	28.7%	0.59 (NS)
Positive	345	65.6%	238	69.0%	107	31.0%	
PR status							
Negative	254	48.3%	181	71.3%	73	28.7%	0.47 (NS)
Positive	272	51.7%	186	68.4%	86	31.6%	
ERBB2 status							
Negative	395	75.1%	279	70.6%	116	29.4%	0.46 (NS)
Positive	131	24.9%	88	67.2%	43	32.8%	
Molecular subtypes							
HR- ERBB2-	101	19.2%	76	75.2%	25	24.8%	0.48 (NS)
HR- ERBB2+	73	13.9%	51	69.9%	22	30.1%	
HR+ ERBB2-	294	55.9%	203	69.0%	91	31.0%	
HR+ ERBB2+	58	11.0%	37	63.8%	21	36.2%	
PIK3CA mutation status [f]							
wild type	354	68.1%	252	71.2%	102	28.8%	0.32 (NS)
mutated	166	31.9%	111	66.9%	55	33.1%	
MKI67 mRNA expression							
median	8.55 (0.22-81.5)		8.43 (0.22-81.5)		8.88 (0.44-71.0)		0.89 (NS) [h]
Metastasis							
No	317	60.3%	224	70.7%	93	29.3%	0.58 (NS)
Yes	209	39.7%	143	68.4%	66	31.6%	

NS: not significant

[a] Chi-squared Test

[b] Scarff Bloom Richardson classification

[c] Information available for 511 patients

[d] Information available for 521 patients

[e] Information available for 516 patients

[f] Information available for 520 patients

[h] Mann-Whitney Test

cancers

MDPI

Table S6. PIWIL1-2-3-4 protein levels of expression in a series of IBCs (*n* = 150) with molecular subtypes.

Protein level	PIWIL1	PIWIL2	PIWIL3	PIWIL4
**Normal tissues (*n* = 10)**	0	Nuclear score: 1-2 (EC), 3 (MC)<br>Cytoplasmic score : 0-0.5	0	Nuclear score: 1-2 (EC, MC)<br>Cytoplasmic score : 0-0.5
**IBCs (*n* = 150)**	N : 98 (65.4%)<br>A : 52 (34.6%)	U : 67 (48.3%)<br>N : 83 (51.7%)	N : 138 (92%)<br>A : 12 (8%)	U : 73 (48.6%)<br>N : 77 (51.4%)
**RH-HER2- (*n* = 22)**	N : 16 (73%)<br>A : 6 (27%)	U : 12 (54%)<br>N : 10 (46%)	N : 21 (95.5%)<br>A : 1 (4.5%)	U : 8 (28.7%)<br>N : 16 (71.3%)
**RH-HER2+ (*n* = 15)**	N : 10 (66.7%)<br>A : 5 (33.3%)	U : 9 (60%)<br>N : 6 (40%)	N : 13 (86.7%)<br>A : 2 (13.3%)	U : 6 (39.7%)<br>N : 9 (60.3%)
**RH+HER2- (*n* = 98)**	N : 66 (67.4%)<br>A : 32 (32.6%)	U : 50 (51%)<br>N : 48 (49%)	N : 92 (93.9%)<br>A : 6 (6.1%)	U : 47 (47.3%)<br>N : 51 (52.7%)
**RH+HER2+ (n = 15)**	N : 12 (80%)<br>A : 3 (20%)	U : 4 (26.6%)<br>N : 11 (73.4%)	N : 11 (73.4%)<br>A : 4 (26.6%)	U : 8 (53.4%)<br>N : 7 (46.6%)

[U: underexpression, O: overexpression, N : normal epression, A : aberrant emerging expression].

Table S7. Percentage of *PIWIL1-3* RNA expression and *PIWIL2-4* RNA underexpression in a multitumor panel.

**Tumors**	**Number**	***PIWIL1* over expression (%) ***	***PIWIL2* under expression (%) ****	***PIWIL3* over expression (%) ***	***PIWIL4* under expression (%) ****
Anal canal	48	12.5	33.3	0.0	83.3
Head and neck	50	8.0	60.0	0.0	12.0
Prostate	48	0.0	25.0	0.0	16.7
Ovary	52	28.8	76.9	0.0	23.1
Thyroid	31	25.8	12.9	0.0	3.2
Cervix	37	27.0	73.0	0.0	27.0
Skin	27	0.0	51.9	3.7	11.1
Endometrium	29	27.6	96.6	0.0	69.0
Colon	49	51.0	32.7	0.0	10.2
Lung	54	1.9	16.7	1.9	9.3
Kidney	22	0.0	27.3	0.0	4.5
Pancreas	22	9.1	9.1	0.0	9.1
Liver	31	0.0	25.8	0.0	32.3
Bladder	49	2.0	85.7	0.0	71.4
Stomach	29	31.0	10.3	0.0	0.0
Brain	50	0.0	14.0	0.0	20.0

* Over expression was defined as Ct < 30.; **Under expression was defined as mRNA expression level <0.33 relative to corresponding normal tissues equal to 1.

Table S8. *Piwil2-4* genes are associated with hallmarks of cancer.

	PIWIL2		***PIWIL4***	
	**r**	**P**	**r**	**P**
**Genome integrity**				
ATM	**0.331**	**<0,0001**	**0.520**	**<0,0001**
ERCC1	0.207	<0,0001	0.084	0.0749
PARP3	0.190	<0,0001	0.117	0.013
XRCC1	0.173	0.0002	0.050	0.2871
BAP1	0.122	0.0101	-0.021	0.652
PARP2	0.058	0.2186	0.038	0.4223
PARP1	0.000	0.9941	-0.011	0.8245
BLM	-0.070	0.1418	0.046	0.3313
BRCA1	-0.084	0.0497	**-0.241**	**<0,0001**
BRCA2	-0.110	0.0105	0.030	0.4865
**Cell cycle**				
p15/CDKN2A	0.183	0.0001	0.191	<0,0001
p16/CDKN2A	0.063	0.1865	0.119	0.012
p19/ARF	-0.011	0.8122	0.050	0.2902
MKI67	-0.120	0.0052	-0.105	0.0148
NEK2	-0.145	0.0021	-0.154	0.0011
PLK1	**-0.184**	**<0,0001**	**-0.183**	**<0,0001**
AURKA	**-0.191**	**<0,0001**	-0.122	0.0099
**RTK signaling**				
PDGFRA	**0.511**	**<0,0001**	**0.558**	**<0,0001**

DDR2	**0.490**	**<0,0001**	**0.573**	**<0,0001**
PDGFRB	**0.469**	**<0,0001**	**0.319**	**<0,0001**
IGF2	**0.461**	**<0,0001**	**0.387**	**<0,0001**
EGFR	**0.375**	**<0,0001**	**0.563**	**<0,0001**
CSF1R	0.276	<0,0001	**0.341**	**<0,0001**
MET	0.247	<0,0001	**0.401**	**<0,0001**
ALK	0.229	<0,0001	0.197	<0,0001
TGFB1	0.204	<0,0001	0.182	<0,0001
IGF2R	0.120	0.0112	0.105	0.0259
RON	0.069	0.1445	0.015	0.755
ERBB2	0.063	0.1469	-0.019	0.6674
DDR1	0.056	0.1918	-0.104	0.0157
IGF1R	0.027	0.5745	**-0.224**	**<0,0001**
ROS	0.002	0.9702	0.099	0.0372
RET	-0.055	0.2462	**-0.197**	**<0,0001**
**MAPK signaling**				
ETV5	**0.348**	**<0,0001**	**0.398**	**<0,0001**
ETV1	**0.327**	**<0,0001**	**0.313**	**<0,0001**
ETV4	0.087	0.0676	0.111	0.0187
**PI3K signaling**				
AKT3	**0.429**	**<0,0001**	**0.552**	**<0,0001**
FOXO1	**0.428**	**<0,0001**	**0.514**	**<0,0001**
FOXO4	**0.359**	**<0,0001**	0.259	<0,0001
PIK3R1	**0.318**	**<0,0001**	0.290	<0,0001
IRS2	**0.313**	**<0,0001**	**0.309**	**<0,0001**
FOXO3	0.283	<0,0001	0.199	<0,0001
PIK3CA	0.277	<0,0001	**0.405**	**<0,0001**
PTEN	0.219	<0,0001	0.246	<0,0001
INPP4B	0.168	<0,0001	-0.011	0.8029
FOXO6	0.142	0.0009	-0.013	0.7697
AKT2	0.123	0.0092	0.085	0.0739
PRKCB	0.068	0.1493	0.298	<0,0001
AKT1	0.064	0.177	-0.019	0.6936
P70S6K	0.012	0.8079	0.022	0.6408
**EMT**				
VIM	**0.421**	**<0,0001**	**0.514**	**<0,0001**
TWIST1	**0.353**	**<0,0001**	**0.389**	**<0,0001**
JUB	**0.318**	**<0,0001**	**0.302**	**<0,0001**
CDH1	-0.045	0.3449	-0.096	0.0421
KRT18	-0.060	0.1646	**-0.281**	**<0,0001**
**Cell migration**				
EHD2	**0.462**	**<0,0001**	**0.310**	**<0,0001**
CAV1	**0.449**	**<0,0001**	**0.446**	**<0,0001**
CDH11	**0.347**	**<0,0001**	0.251	<0,0001
NCAM1	**0.345**	**<0,0001**	**0.457**	**<0,0001**
ITGB5	0.292	<0,0001	0.126	0.0074
HAS2	0.279	<0,0001	**0.419**	**<0,0001**
SERPINE2	0.276	<0,0001	**0.458**	**<0,0001**
ITGA5	0.254	<0,0001	0.188	<0,0001
MMP14	0.228	<0,0001	0.175	<0,0001

HAS3	0.154	0.0004	0.290	<0,0001
PLAU	0.139	0.0033	0.132	0.005
CDH3	0.112	0.009	**0.351**	**<0,0001**
IGFBP5	0.110	0.0202	0.050	0.2913
CD44	0.095	0.0295	0.269	<0,0001
NME4	0.084	0.0522	-0.116	0.0072
NME2	0.084	0.0525	0.020	0.6388
HAMP	0.047	0.3196	0.075	0.1148
MMP13	0.004	0.9386	0.010	0.8309
NME1	-0.156	0.0003	**-0.250**	**<0,0001**
**Stemness**				
ALDH1A1	**0.361**	**<0,0001**	**0.460**	**<0,0001**
ALDH1A3	**0.336**	**<0,0001**	**0.482**	**<0,0001**
CD133	0.198	<0,0001	**0.391**	**<0,0001**
ASCL1	0.000	0.9998	-0.036	0.4509
HTERT	-0.062	0.1891	-0.032	0.5031
**Angiogenesis**				
VEGFC	**0.419**	**<0,0001**	**0.481**	**<0,0001**
PTGS2	**0.319**	**<0,0001**	**0.527**	**<0,0001**

© 2020 by the authors. Submitted for possible open access publication under the terms and conditions of the Creative Commons Attribution (CC BY) license (http://creativecommons.org/licenses/by/4.0/).
